# A network meta-analysis of pedagogical models in physical education: evaluating multidimensional learning outcomes and instructional duration effects

**DOI:** 10.3389/fpsyg.2026.1766890

**Published:** 2026-03-03

**Authors:** Guo Jingxia, Yu Qichao, Zulezwan bin Ab Malik

**Affiliations:** 1 Faculty of Sport Science and Coaching, Universiti Pendidikan Sultan Idris (UPSI), Tanjung Malim, Perak, Malaysia; 2 Faculty of Physical Education, Hanjiang Normal University, Shiyan, Hubei, China

**Keywords:** Bayesian network meta-analysis, hybrid pedagogical model, learning outcomes, pedagogical models, physical education

## Abstract

**Purpose:**

This study systematically evaluated the comparative effectiveness of pedagogical models (PMs) on multidimensional learning outcomes in physical education and examined the moderating role of instructional duration.

**Methods:**

A systematic search was conducted across seven databases (e.g., Web of Science, PubMed) for studies published between 2000 and 2025. Methodological quality was assessed using MINORS. A Bayesian Network Meta-Analysis (NMA) was performed using standardized mean differences (SMDs) to enable comparison across diverse outcome measures.

**Results:**

Sixty-five studies met the inclusion criteria. Probabilistic rankings suggested that Hybrid Pedagogical Models (HPMs) showed comparatively high effectiveness for tactical decision-making (SUCRA = 80.1%) and skill execution (SUCRA = 77.2%), Teaching Games for Understanding (TGfU) ranked highest for student motivation (SUCRA = 82.1%), and Sport Education (SE) demonstrated favourable effects for enjoyment (SUCRA = 74.0%). However, pairwise comparisons revealed overlapping credible intervals, indicating uncertainty in the magnitude of differences between models. In addition, significant publication bias was detected for skill execution outcomes (*p* = 0.0077), suggesting that these effects should be interpreted cautiously. Subgroup analyses identified instructional duration as an important moderator, with interventions approaching or exceeding approximately 720 min—equivalent to a sustained instructional unit—associated with more consistent effects and reduced heterogeneity.

**Conclusion:**

No single pedagogical model can be considered universally superior. Instead, the findings support a goal-oriented approach to model selection aligned with intended learning outcomes and implemented over sufficient instructional duration. Ensuring sustained exposure (≈720 min) appears critical for enabling student-centered pedagogies to realize their educational potential.

## 1 Introduction

In the context of global educational transformation, physical education (PE) is no longer viewed merely as a vehicle for physical training but is increasingly recognised as a central platform for whole-person education, contributing to students’ cognitive development, motor competence, emotional regulation, social responsibility, and lifelong health literacy. However, a persistent challenge remains: how can educators strategically select instructional approaches to achieve specific, multidimensional learning goals? Traditional PE practices, which predominantly rely on repetitive skill drills, have proven inadequate in this regard. They emphasize short-term skill mastery at the expense of broader cognitive, emotional, and social development (Fernandez-Rio and Iglesias, 2024), often failing to effectively engage students.

To address these limitations, student-centred pedagogical models (PMs) grounded in constructivist theories have been promoted. These models advocate for active, meaningful learning experiences. Key examples include Sport Education (SE), which fosters authentic sporting experiences and team engagement through role responsibilities (Siedentop, 1994). Teaching Games for Understanding (TGfU) prioritizes tactical understanding within realistic game scenarios (Bunker and Thorpe, 1982). Cooperative Learning (CL) emphasizes peer cooperation and reciprocal teaching, enhancing collective cognitive and skill-based learning (Casey and Goodyear, 2015), while Teaching Personal and Social Responsibility (TPSR) develops social–emotional skills and responsible behaviours through structured accountability (Hellison, 2010). Collectively, these models reflect a shift toward learner-centred, context-based pedagogies designed to address multiple domains of student development simultaneously. However, their pedagogical diversity, while theoretically advantageous, creates practical uncertainty for teachers attempting to align specific instructional goals with an appropriate model.

Yet, the very proliferation of these models presents a new dilemma for practitioners: selection. Each model exhibits inherent strengths but also limitations. For instance, TGfU often lacks explicit skill instruction (Psotta and Martin, 2011), whereas the complexity intrinsic to SE can reduce engagement among some students (Perlman, 2012). As PE curricula increasingly require educators to achieve multiple learning objectives within limited instructional time, the challenge shifts from identifying effective models to determining which model, or combination of models, is most suitable for specific learning goals and teaching contexts. This has led to a conceptual impasse – if no single model is universally superior, on what basis should educators choose? In response to this practical decision-making challenge, Hybrid Pedagogical Models (HPMs) have emerged as a promising strategy, intentionally integrating complementary pedagogical features to maximise learning effectiveness across domains. Empirical studies suggest their promise; for example, integrating TGfU with SE enhances tactical decision-making (Pan et al., 2023), and combining CL and TGfU fosters emotional growth and collaboration (Ben Khalifa et al., 2020).

Despite this rich landscape, comparative guidance remains sparse. Qualitative reviews have charted the field: González-Víllora et al. (2019) introduced a descriptive framework for HPMs, while Fernandez-Rio and Iglesias (2024), in an umbrella review, confirmed positive outcomes of PMs but underscored the influence of critical contextual moderators like instructional duration. These reviews have provided valuable conceptual mapping and general effectiveness summaries; however, they did not generate quantitative comparative rankings across models, nor did they statistically evaluate instructional duration as a continuous moderator influencing model effectiveness. Consequently, existing evidence cannot inform evidence-based model selection, because it lacks both cross-model comparability and statistical examination of instructional dosage effects.

Two critical gaps remain unaddressed. First, existing evidence is fragmented, lacking a unified statistical framework to enable simultaneous comparison through a network-based framework the relative effectiveness of all PMs and HPMs across diverse learning domains. This leaves educators without a clear, evidence-based map for model selection. Second, while instructional duration is widely acknowledged as influential, its role as a potential moderator of model effectiveness has not been systematically quantified alongside model type. It remains unclear whether the superiority of a given model is contingent upon receiving a sufficient “dosage” of instruction.

To bridge these gaps, this study introduces a goal-oriented framework for model selection in PE. We employ a Bayesian Network Meta-Analysis (NMA) to estimate the relative effectiveness of major PMs and HPMs within a connected evidence network against common comparators across key cognitive, psychomotor, and affective learning outcomes; and (2) to quantitatively investigate how instructional duration and sport type moderate model effectiveness. By integrating quantitative comparative ranking with moderator modelling, this study provides an evidence-based decision framework that helps educators align pedagogical model selection with specific learning goals and contextual teaching conditions. By simultaneously evaluating which models demonstrate greater suitability for specific learning outcomes and identifying the critical conditions for efficacy, this research moves beyond the quest for a single best model and offers an evidence-informed framework to support pedagogical decision-making for designing sustainable, context-sensitive PE instruction.

## 2 Methods

### 2.1 Data sources and study selection

This systematic review adhered to the PRISMA guidelines (Page et al., 2021) and conducted comprehensive searches in various databases, including Google Scholar, Web of Science, PubMed, Elsevier, Scopus, CNKI, and Wanfang, covering the literature from January 2000 to June 2025. The search utilized a structured strategy with Boolean operators and keywords: TS = ((“sport education” OR “cooperative learning” OR “teaching games for understanding” OR “tactical games” OR “game-based approach” OR “tactical games approach” OR “tactical approach” OR “tactical games model” OR “teaching for personal and social responsibility” OR “hybrid pedagogical model” OR “pedagogical model”) AND (“sport” OR “physical education” OR “training”) AND (“decision making” OR “skill execution” OR “motivation” OR “enjoyment”) AND (“intervention” OR “experimental” OR “quasi-experimental” OR “randomized controlled trial”)). The full electronic search strategy for Web of Science is provided in Supplementary material S1 to ensure reproducibility. Spain has a large body of literature related to pedagogical models, so only English, Spanish or Chinese articles were searched. The review protocol was not pre-registered in PROSPERO or other registries. This is acknowledged as a methodological limitation.

### 2.2 Inclusion and exclusion criteria

The study applied the PICOS framework (Population, Intervention, Comparison, Outcomes, and Study design) with specific criteria: (1) Population: Students and athletes without pre-existing injuries or medical conditions. (2) Intervention: The experimental group employed established teaching methods, including physical education, cooperative learning, teaching games for understanding, and teaching personal and social responsibility, or a combination of these methods. (3) Comparison: The control group employed traditional teaching approaches. (4) Outcomes: Evaluation of tactical ability (decision making), technical skills (skill execution), and emotional aspects (motivation and enjoyment). (5) Study Design: The included studies primarily employed quasi-experimental designs, given the structured nature of educational interventions, such as fixed class assignments. Exclusion criteria encompassed: (1) Self-controlled study designs; (2) Reviews, theses, or dissertations; (3) Studies lacking complete or sufficient data for outcome extraction.

### 2.3 Literature screening and data extraction

All identified records were de-duplicated using EndNote X9. Two independent reviewers conducted a double-blind screening of titles, abstracts, and keywords according to the eligibility criteria, followed by full-text assessment. Inter-rater reliability was evaluated using Cohen’s Kappa (*κ* = 0.85), indicating strong agreement. Data extraction focused on: (1) General Information: First author, publication year, and study characteristics. (2) Intervention Characteristics: Study design, sample size, participant demographics (including age and gender), teaching content, and duration of the intervention. (3) Outcome Measures: Tactical ability (“decision-making”), technical ability (“skill execution”), and emotional outcomes (“motivation” and “enjoyment”).

Because primary studies reported intervention length using heterogeneous units (e.g., lessons, weeks, or months), instructional duration was standardized into total instructional minutes using a predefined conversion protocol to enable quantitative comparison of intervention “dosage.”

The following rules were applied: (1) When weekly frequency and session length were reported: Total minutes = sessions/week × minutes/session × number of weeks. (2) When duration was reported in months: Months were converted assuming 4 weeks per month, followed by the same calculation. (3) When only the number of weeks was reported without session details: A standardized exposure of 90 min/week (two 45-min PE lessons) was assumed. (4) When duration was described only qualitatively (e.g., “one semester”): The intervention was conservatively classified as ≥ 720 min, representing the minimum exposure expected in a typical semester-based PE curriculum.

This harmonization procedure allowed consistent subgroup classification while applying identical assumptions across studies to maintain comparability.

### 2.4 Quality evaluation

The methodological quality of the included studies was assessed using the Methodological Index for Non-Randomized Studies (MINORS), which evaluates 12 methodological aspects. Each domain was scored as 0 (not reported), 1 (reported but inadequate), or 2 (reported and adequate), with a maximum score of 24. Two reviewers independently scored each study; discrepancies were resolved by discussion. Inter-rater reliability for MINORS scoring was also high (*κ* = 0.89).

### 2.5 Statistical analysis

The validity of indirect comparisons in the network meta-analysis was based on the assumption of transitivity. Clinical and methodological comparability across studies was evaluated in terms of participant characteristics (age group and educational setting), intervention implementation (school-based PE programs), outcome constructs, and comparator conditions (primarily traditional instruction).

All analyses were conducted using R Studio (version 4.4.3) and Stata 15.1 (StataCorp, TX, USA). Bayesian Network Meta-Analysis (NMA) was performed in R using the gemtc (version 0.8–2) and rjags (version 4–13) packages, with data management via dplyr. Stata 15.1 was used for subgroup analyses.

Continuous outcomes were summarized as Standardized Mean Differences (SMDs) with 95% Credible Intervals (CrIs). A Bayesian random-effects model was applied to account for between-study variability, using non-informative priors to minimize prior influence on posterior estimates.

Markov Chain Monte Carlo (MCMC) sampling was conducted with four chains for 50,000 iterations, with the first 20,000 iterations discarded as burn-in. Convergence was assessed using the Potential Scale Reduction Factor (PSRF), with values approaching 1.0 indicating convergence. Trace plots and posterior density plots were visually inspected to confirm adequate chain mixing.

Local inconsistency was evaluated using node-splitting analyses comparing direct and indirect evidence, with *p* > 0.05 indicating no significant inconsistency. The ranking of pedagogical models was estimated using the Surface Under the Cumulative Ranking curve (SUCRA), ranging from 0% (least effective) to 100% (most effective).

Publication bias was assessed through comparison-adjusted funnel plots generated using the netmeta package and quantified using Egger’s regression test.

Between-study heterogeneity was examined using both conventional I^2^ statistics and the posterior median of the between-study standard deviation (*τ*) with corresponding 95% CrIs. To explore potential sources of variability, subgroup analyses stratified by instructional duration and sport type were conducted using random-effects models in Stata 15.1.

## 3 Results

### 3.1 Literature screening

**Figure 1:**
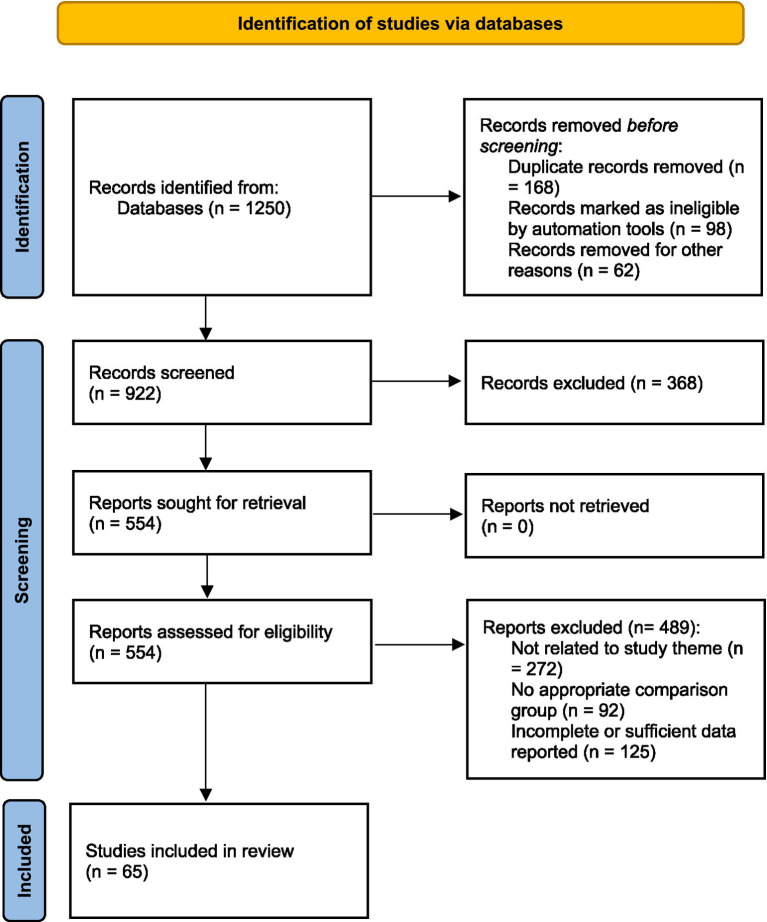
PRISMA 2020 flow diagram of the study selection process.

A comprehensive search across seven databases identified 1,250 articles. After removing duplicates, 922 articles remained. Title and abstract screening excluded 368 articles, and 489 were excluded after full-text review. Ultimately, 65 articles met the inclusion criteria and were included in the analysis. The study selection process is illustrated using a PRISMA 2020 flow diagram (Figure 1).

### 3.2 Characteristics of included studies

Disagreements during the screening process were resolved through discussions between two independent researchers. A total of 65 quasi-experimental studies (pre- and post-test) published in English were included, encompassing 8,027 participants across 16 countries. The interventions assessed various pedagogical models, including SE, TGfU, CL, TPSR, and HPMs, in comparison to traditional teaching methods. Geographically, the majority of studies were conducted in Europe (63.0%, primarily in Spain), followed by Asia (18.5%) and North America (10.8%). Participants ranged from K-12 to higher education, with middle school students constituting the largest subgroup at 47.4%. The duration of interventions varied, with the most common range being 720–1,440 min (37.0%). Detailed characteristics of the interventions and outcome measures are summarized in Table 1 and Supplementary Table S1.

**Table 1:** Characterizing the distribution of included studies.

Category	Studies, *n* (%)	Participants, *n* (%)
Sample and model distribution		
TGfU	18 (27.7%)	1,192 (14.8%)
SE	22 (33.8%)	3,165 (39.4%)
CL	5 (7.7%)	983 (12.2%)
TPSR	8 (12.3%)	1,499 (18.7%)
HPMs	12 (18.5%)	1,188 (14.8%)
Geographical distribution		
Europe	41 (63.0%)	5,410 (67.4%)
Asia	12 (18.5%)	916 (11.4%)
North America	7 (10.8%)	1,355 (16.9%)
Oceania	3 (4.6%)	271 (3.4%)
Africa	2 (3.1%)	75 (0.9%)
Educational levels of participants		
Primary school	12 (18.5%)	1,664 (20.7%)
Middle school	29 (44.6%)	3,801 (47.4%)
High school	11 (16.9%)	1,398 (17.4%)
College	9 (13.8%)	1,065 (13.3%)
Experienced	4 (6.2%)	99 (1.2%)
Intervention duration		
<720 min	19 (29.2%)	1,654 (20.6%)
720-1440 min	28 (43.1%)	2,971 (37.0%)
>1,440 min	12 (18.5%)	2,283 (28.4%)
Not reported	6 (9.2%)	1,119 (13.9%)
Quality evaluation		
Low quality	4 (6.2%)	207 (2.6%)
Medium quality	26 (40.0%)	3,617 (45.0%)
High quality	35 (53.8%)	4,203 (52.4%)

### 3.3 Quality evaluation

The quality of the included studies was assessed using the Methodological Index for Non-Randomized Studies (MINORS) tool, with the results summarized in Supplementary Table S2. Of the 65 studies, 35 (53.8%) were rated as high quality (scores: 19–24), representing a total sample of 4,203 participants (52.4%). Twenty-six studies (40.0%) were classified as medium quality (scores: 16–18), with a combined sample of 3,617 participants (45.0%). The remaining four studies (6.2%) were considered low quality (scores: 13–15), involving 207 participants (2.6%). Studies receiving lower scores were typically characterized by small sample sizes, non-equivalent baselines, lack of blinding procedures, and incomplete reporting of dropout rates (Andrianto, 2023; Wallhead and Ntoumanis, 2004).

### 3.4 Network meta-analysis

**Figure 2:**
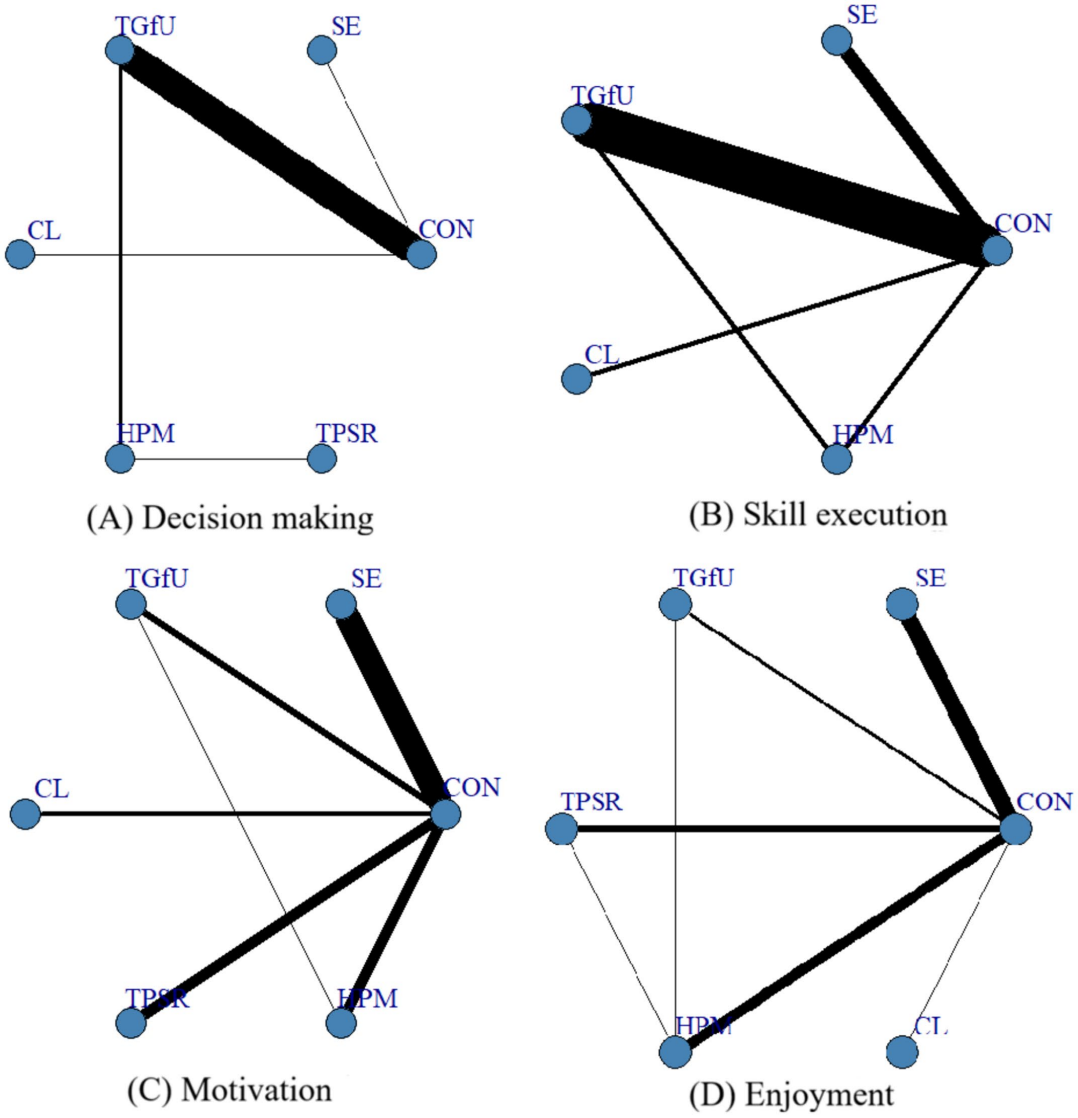
Network diagram of the relationship between different pedagogical models on learning outcomes. **(A)** Decision making; **(B)** Skill execution; **(C)** Motivation; **(D)** Enjoyment.

A total of 65 papers were included in this study. As shown in Figure 2, the thickness of the connecting lines in the network plot indicates the number of corresponding studies, i.e., the thicker the line, the more comparative studies between the two groups of interventions. In all the literature, the frequency of the different outcome indicators was as follows: decision-making was used in 18 studies with 23 data sets; skill execution was used in 26 studies with 44 data sets; and motivation was used in 33 studies with 37 data sets; Enjoyment was used in 22 studies with 26 data sets.

Figure 2 illustrates that, among the aforementioned outcome indicators, most studies have focused on direct comparisons between instructional models and control groups. In contrast, the number of two-by-two comparisons between different instructional models was relatively small. Based on the thickness of the connecting lines in the network plot, it can be further observed that, for the decision-making and skill execution indicators, more studies were comparing TGfU with the control group, whereas for the motivation and enjoyment indicators, the number of studies comparing SE with the control group was the number of studies on motivation and enjoyment between SE and the control group was more significant.

### 3.5 Consistency assessment and model convergence

The validity of the network meta-analysis depends on the assumption of consistency between direct and indirect evidence. Comparison of the Deviance Information Criterion (DIC) between the consistency model and the unrelated mean effects (UME) model showed negligible differences (< 5 points) across all outcomes, indicating no significant inconsistency within the network. Accordingly, the consistency model was adopted for all analyses.

Model convergence was evaluated using the Potential Scale Reduction Factor (PSRF), with all parameters stabilizing at 1.00, indicating satisfactory convergence of the Markov chains. Visual inspection of trace plots demonstrated stable sampling without drift, and posterior density plots showed smooth, unimodal distributions, confirming adequate chain mixing and posterior stability (Supplementary Figure S1).

### 3.6 Local consistency check

To further validate the network structure, node-splitting analysis was performed to evaluate local inconsistency by comparing direct and indirect evidence within closed loops. The analysis revealed no significant discrepancies (*p* > 0.05) between direct and indirect estimates for any comparison, supporting the assumption of consistency underlying the network model. Detailed results of the node-splitting assessment are provided in Supplementary Table S3.

### 3.7 Direct meta-analysis comparison

**Figure 3:**
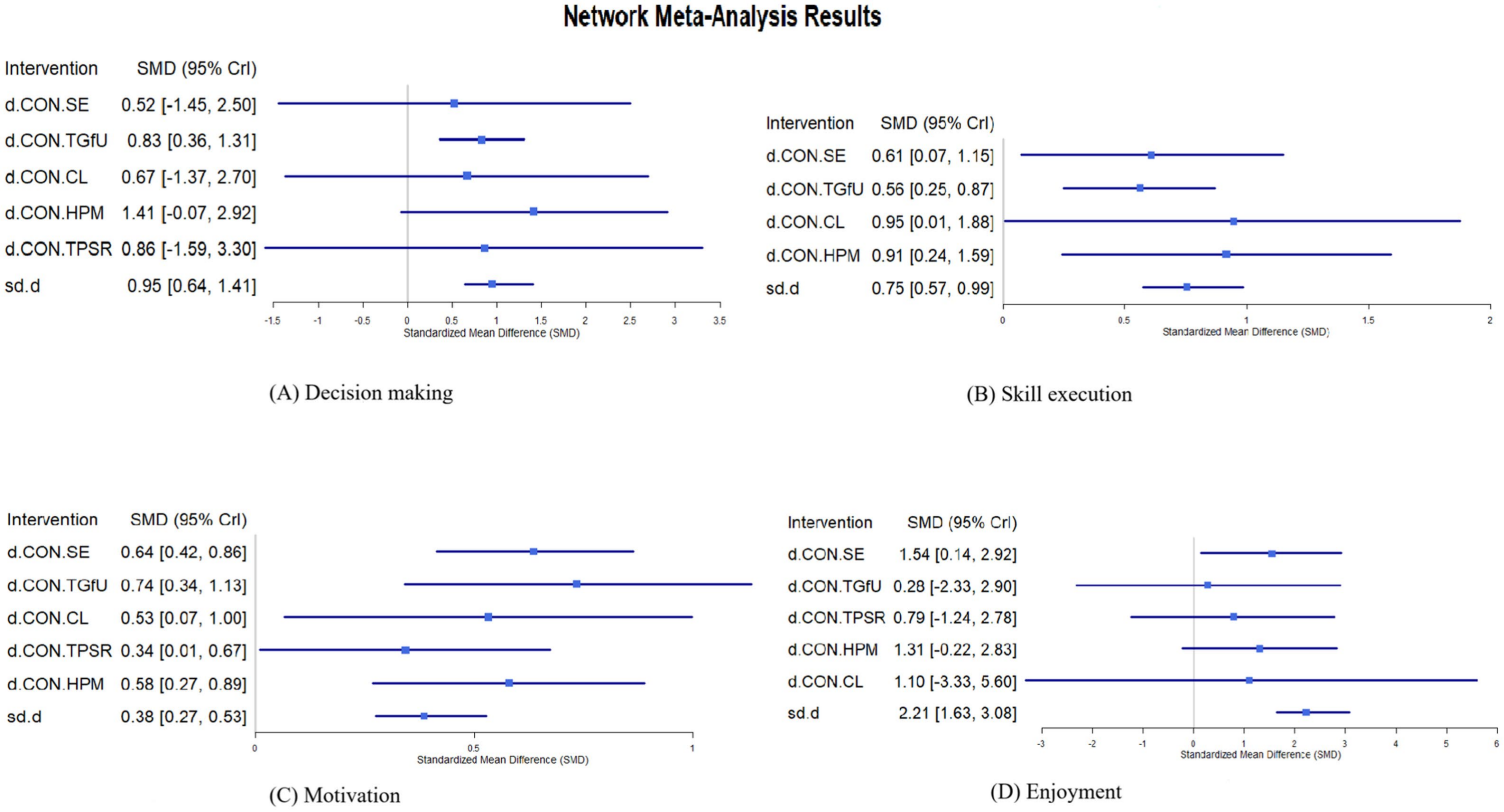
Forest plots showing standardized mean differences (SMDs) with 95% credible intervals for the four outcomes in the network meta-analysis. **(A)** Decision making; **(B)** Skill execution; **(C)** Motivation; **(D)** Enjoyment.

1. Forest plots were generated to analyze direct comparisons of teaching models for each outcome indicator: 1) Decision Making: No significant differences were observed for SE [SMD = 0.52, 95%CrI: (−1.45, 2.50)], TGfU [SMD = 0.83, 95%CrI: (0.36, 1.31)], CL [SMD = 0.67, 95%CrI (−1.37, 2.70)], HPM [SMD = 1.41, 95%CrI: (−0.07, 2.92)] and TPSR [SMD = 0.86, 95%CrI (−1.59, 3.30)] (Figure 3A).
2. Skill Execution: TGfU showed a positive estimated effect over controls SE [SMD = 0.61, 95%CrI: (0.07, 1.15)], TGfU [SMD = 0.56, 95%CrI: (0.25, 0.87)] and CL [SMD = 0.95, 95%CI (0.01, 1.88)]. No significant differences were found for HPM [SMD = 0.91, 95%CrI: (0.24, 1.59)] (Figure 3B).
3. Motivation: SE [SMD = 0.64, 95%CrI: (0.42, 0.86)], TGfU [SMD = 0.74, 95%CrI: (0.34, 1.13)], CL [SMD = 0.53, 95%CrI: (0.07, 1.00)], TPSR [SMD = 0.34, 95%CrI: (0.01, 0.67)] and HPM [SMD = 0.58, 95%CrI: (0.27, 0.89)] outperformed controls (Figure 3C).
4. Enjoyment: SE [SMD = 1.54, 95%CrI: (0.14, 2.92)], significantly improved enjoyment compared to controls. No significant differences were observed for TGfU [SMD = 0.28, 95%CrI: (−2.33, 2.90)], TPSR [SMD = 0.79, 95%CrI: (−1.24, 2.78)], HPM [SMD = 1.31, 95%CrI: (−0.22, 2.83)], CL [SMD = 1.10, 95%CrI: (−3.33, 5.60)] (Figure 3D).

### 3.8 Cumulative probability ranking results

The rankings reflect the relative probability of each model being the most effective in improving each specific learning outcome. Surface Under the Cumulative Ranking (SUCRA) values were used to rank pedagogical models for each outcome (Table 2). 1) Decision making: HPM (80.1%) > TGfU (57.1%) > TPSR (54.5%) > CL (49.6%) > SE (43.0%) > CON (15.9%). 2) Skill execution: HPM (77.2%) > CL (75.5%) > SE (51.2%) > TGfU (45.0%) > CON (1.1%). 3) Motivation: TGfU (82.1%) >SE (70.7%) >HPM (61.3%) > CL (55.0%) > TPSR (30.2) > CON (0.7%). 4) Enjoyment: SE (74.0%) > HPM (66.9%) > CL (55.2%) > TPSR (49.0%) > TGfU (35.0%) > CON (1.2%).

**Table 2:** SUCRA ranking probabilities of pedagogical models across learning outcomes.

Pedagogical model	Decision making	Skill execution	Motivation	Enjoyment
CON	15.9%	1.1%	0.7%	12.0%
SE	43.0%	51.2%	70.7%	74.0%
TGfU	57.1%	45.0%	82.1%	35.0%
CL	49.6%	75.5%	55.0%	55.2%
HPM	80.1%	77.2%	61.3%	66.9%
TPSR	54.5%	NA	30.2%	49.0%

### 3.9 Pairwise comparisons across outcomes

To complement SUCRA rankings, pairwise relative effects between pedagogical models were examined using Bayesian league tables (Supplementary Tables S4–S7). Across all outcomes, most comparisons were associated with wide and overlapping 95% credible intervals (CrIs), indicating considerable uncertainty in the magnitude of differences between interventions.

For decision-making, Teaching Games for Understanding (TGfU) showed a positive effect compared with traditional instruction (CON) (SMD = 0.83, 95% CrI 0.36–1.31), whereas comparisons among active pedagogical models demonstrated overlapping CrIs. For skill execution, several models produced favourable estimates relative to CON, but nearly all inter-model contrasts included the null value. For motivation and enjoyment, CrIs for all pairwise comparisons spanned zero, suggesting similar estimated effects across pedagogical approaches.

### 3.10 Publication bias analysis

For the 65 included studies, the risk of publication bias was assessed in this study, and the results are shown in Supplementary Figure 1. Based on the funnel plot and Egger’s test results (derived using RStudio 4.4.3 and rjags), the distribution of effect sizes appeared to be concentrated in the upper region of the funnel plot. This pattern suggests that most studies had relatively large sample sizes. Only a few studies were located at the bottom or near the edges of the funnel, indicating a small-study effect. Egger’s test further supported this observation: the *p*-values for decision-making (*p* = 0.1582), motivation (*p* = 0.2101), and enjoyment (*p* = 0.0245) were all above the 0.05 threshold, indicating no significant evidence of publication bias for these outcomes.

However, for skill execution, Egger’s test revealed a statistically significant result (*p* = 0.0077), suggesting potential publication bias in this domain. Consequently, the findings related to skill execution should be interpreted with caution, and future research should prioritize high-quality, well-reported studies to verify and strengthen the current evidence base.

### 3.11 Heterogeneity and subgroup analysis

Between-study heterogeneity was quantified using the posterior median of the between-study standard deviation (*τ*) with 95% credible intervals (CrIs) (Table 3).

**Table 3:** Estimated between-study heterogeneity.

Outcome	τ (Median)	95% credible interval
Decision-Making	4.97	3.28–7.97
Skill Execution	4.97	3.27–7.93
Motivation	4.97	3.28–7.86
Enjoyment	2.17	1.63–3.07

For decision-making, skill execution, and motivation, τ values were similar (median τ = 4.97), with wide CrIs (Decision-making: 3.28–7.97; Skill execution: 3.27–7.93; Motivation: 3.28–7.86). Enjoyment showed comparatively lower heterogeneity (median τ = 2.17, 95% CrI 1.63–3.07).

Conventional heterogeneity statistics from pairwise analyses were consistent with these findings, with I^2^ values generally exceeding 70% (Supplementary Table S8).

Subgroup analyses were conducted in Stata 15.1 using random-effects models stratified by instructional duration and sport type (Table 4). Interventions exceeding approximately 720 min tended to show larger effect estimates than shorter-duration interventions (<720 min). Differences between team and individual sport contexts were also observed in selected outcomes, although estimates were imprecise in analyses with limited studies.

**Table 4:** Subgroup analysis examining the moderating effects of instructional duration and sport type on learning outcomes.

Outcome	Category	Heterogeneity		SMD and 95%CI	Effect test		Studies (*n*)
		I^2^	*P*		*Z*	*P*	
Decision making							
TGfU vs. CON	≤720 min	84.0%	<0.001*	0.45 (0.28, 0.62)	5.11	0.000*	11
	720 min-1440 min	26.1%	0.239	0.95 (0.62, 1.29)	5.54	0.000*	6
	>1,440 min	0.0%	<0.001*	2.20 (1.58, 2.82)	6.99	0.000*	1
	Team sports	83.8%	<0.001*	0.68 (0.53, 0.83)	8.71	0.000*	17
	Individual sports	0.0%	<0.001*	0.09 (−0.60, 0.78)	0.25	0.800	1
Skill execution							
TGfU vs. CON	≤720 min	52.2%	0.018*	0.01 (−0.16, 0.17)	0.092	0.927	12
	720 min-1440 min	83.1%	<0.001*	0.72 (0.52, 0.92)	7.13	0.000*	11
	>1,440 min	0.0%	0.526	1.09 (0.80, 1.39)	7.19	0.000*	4
	Team sports	81.5%	<0.001*	0.39 (0.27, 0.51)	6.26	0.000*	25
	Individual sports	72.3%	0.057	0.80 (0.38, 1.21)	3.73	0.000*	2
Motivation							
SE vs. CON	≤720 min	87.7%	<0.001*	0.52 (0.39, 0.65)	7.79	0.000*	7
	720 min-1440 min	77.6%	<0.001*	0.42 (0.29, 0.56)	6.23	0.000*	7
	>1,440 min	0.0%	<0.001*	0.37 (0.21, 0.53)	4.49	0.000*	1
	Team sports	84.5%	<0.001*	0.58 (0.46, 0.69)	9.91	0.000*	10
	Mixed	68.0%	0.025*	0.31 (0.20, 0.43)	5.25	0.000*	4
	Individual sports	0.0%	<0.001*	0.40 (−0.18, 0.98)	1.34	0.180	1
Enjoyment							
SE vs. CON	≤720 min	96.2%	<0.001*	0.37 (0.22, 0.53)	4.72	0.000*	4
	720 min-1440 min	90.1%	<0.001*	0.28 (0.12, 0.45)	3.32	0.001*	3
	>1,440 min	99.6%	<0.001*	0.98 (0.83, 1.12)	13.57	0.000*	5
	Team sports	91.0%	<0.001*	0.50 (0.38, 0.62)	7.94	0.000*	9
	mixed	99.8%	<0.001*	0.68 (0.55, 0.80)	10.44	0.000*	3
HPM vs. CON	≤720 min	98.5%	<0.001*	0.93 (0.67, 1.19)	7.019	0.000*	3
	720 min-1440 min	17.0%	0.272	1.38 (0.96, 1.80)	6.45	0.000*	2
	>1,440 min	0.0%	0.569	0.96 (0.72, 1.20)	7.74	0.000*	2
	Team sports	76.1%	<0.001*	0.80 (0.64, 0.97)	9.39	0.000*	6
	Individual	0.0%	<0.001*	5.04 (4.31, 5.78)	13.43	0.000*	1

Note, in this table, the subgroups are organized by outcome type, category (including time duration and sport type), heterogeneity test results (I^2^ and P-values), and statistical measures (SMD, CI, Z, and *P*-values). *indicates statistical significance at *p* < 0.05.

## 4 Discussion

### 4.1 Beyond the “panacea”: the paradigm of specialized effectiveness

The included studies predominantly employed quasi-experimental designs, focusing largely on K–12 and college students, with research heavily concentrated in Europe. The most significant contribution of this network meta-analysis is its definitive rejection of the quest for a universal pedagogical model. Instead, our findings establish a new paradigm of specialized effectiveness. While traditional PE often relies on a one-size-fits-all approach, this study demonstrates that pedagogical models act as specialized tools for distinct educational domains.

However, this differentiation should be interpreted as probabilistic rather than definitive, as the league table analyses revealed substantial overlap in credible intervals between models, indicating uncertainty in the magnitude of these differences.

Hybrid Pedagogical Models (HPMs) appear particularly suited to supporting tactical cognition and decision-making, Cooperative Learning (CL) shows potential for facilitating technical skill development, Sport Education (SE) demonstrates strong alignment with motivational outcomes, and Teaching Personal and Social Responsibility (TPSR) contributes meaningfully to enjoyment and emotional engagement.

These patterns should not be interpreted as strict hierarchies of effectiveness but rather as tendencies observed within a network largely informed by indirect comparisons with traditional instruction.

This findings suggest a shift in professional practice: from “model adherence,” where instruction is constrained by a single pedagogical framework, to “goal-oriented selection,” where the specific learning objective guides pedagogical choice.

### 4.2 Unpacking the “why”: theoretical mechanisms of domain- specific superiority

To move beyond empirical correlation and understand the causal logic behind these tendencies, we examine the psycho-pedagogical architecture of the models showing comparatively favourable rankings.

#### 4.2.1 Skill mastery: the scaffolding mechanism in hybrid and cooperative models

Our NMA positioned Hybrid Pedagogical Models (HPMs) and Cooperative Learning (CL) among the highest-ranked approaches for skill execution (SUCRA = 77.2 and 75.5%). Yet, pairwise comparisons demonstrated wide and overlapping credible intervals, meaning that statistical superiority over other models could not be established with certainty. Egger’s test revealed significant publication bias (*p* = 0.0077) for skill outcomes, implying that the observed advantages may be overestimated. This bias suggests that studies reporting null or modest effects may be underrepresented in the literature, and therefore the apparent strength of HPM and CL for skill execution.

Despite this statistical caution, the theoretical mechanism underlying these models remains compelling. Mechanistically, the efficacy of CL—and by extension HPMs integrating cooperative structures—is rooted in Vygotskian social constructivism, operationalized through distributed peer scaffolding (Ben Khalifa et al., 2020; Altınkök, 2017).

As observed in empirical studies, verbal interactions and peer debates allow students to provide immediate, contextualized observation, cueing, and error correction during task execution. This process not only increases the frequency of feedback but also engages learners in metacognitive dialogue about movement patterns. The result is a more deeply encoded refinement of motor skills, as learners collaboratively construct and correct performance within their collective Zone of Proximal Development. While Sport Education (SE) proved most effective for enjoyment in this study (SUCRA = 74.0%), the social-interactive engine of CL remains a vital contributor to affective engagement by fulfilling psychological needs for relatedness (Cecchini Estrada et al., 2019; Fernández-Río et al., 2014).

#### 4.2.2 Sport education (SE) and TGfU: affective engagement and intrinsic motivation

Teaching Games for Understanding (TGfU) ranked highest for motivation (SUCRA = 82.1%), while Sport Education (SE) showed favourable probabilities for enjoyment (SUCRA = 74.0%). However, league table comparisons indicated that affective outcomes were characterised by particularly large uncertainty, with nearly all model contrasts exhibiting CrIs that crossed zero.

Theoretically, SE’s dominance in the affective domain is rooted in its capacity to create an autonomy-supportive micro-society. The enjoyment does not stem merely from “fun games,” but from the satisfaction of basic psychological needs—specifically relatedness and autonomy. By sustaining team affiliation over a long-term “season,” SE fosters a profound sense of belonging and identity that transient units cannot replicate. Furthermore, the culmination of the season in a “festive” event celebrates collective improvement rather than raw victory, reducing social anxiety and enhancing the emotional quality of the experience. This combination of social belonging and role-based responsibility explains why SE remains the most powerful driver for student enjoyment, sustaining engagement through deep emotional investment (Viciana et al., 2020; Tendinha et al., 2021).

#### 4.2.3 Hybrid pedagogical models (HPMs): synergistic architecture for cognitive-motor transfer

The comparatively high SUCRA rankings of HPMs for tactical decision-making and skill execution (SUCRA = 80.1 and 77.2%) likely reflect theoretical synergy rather than unequivocal empirical dominance. Given the predominantly star-shaped evidence network, these rankings were derived largely from indirect comparisons, which limits the strength of causal claims regarding model superiority.

Consider the dominant hybrid, SE-TGfU. TGfU alone provides the essential cognitive scaffolding—the questioning strategies and game-form modifications that teach why and when to act. However, without a sustained, meaningful context, this knowledge can remain inert or abstract (Pan et al., 2023). SE provides the applied, situated context—the “season” with its team loyalties, roles, and competitive stakes. By integrating these models, HPMs create a powerful integrative architecture: students learn tactical principles (via TGfU) and immediately consolidate, test, and adapt them within the psychologically authentic pressure of a team season (via SE). This alignment forces the transfer of declarative knowledge into fluid, contextualized motor execution, effectively bridging the gap between tactical awareness (“knowing what to do”) and expert game performance (“doing it under pressure”) (Gil-Arias et al., 2021; Buendía et al., 2022).

### 4.3 The non-negotiable catalyst: instructional duration as a critical moderator

A pivotal finding of this NMA is the identification of instructional duration as a key moderator of efficacy, establishing a clear dose–response relationship. Our subgroup analysis reveals a bifurcation point at 720 min, which serves as a critical threshold for pedagogical impact.

From a practical perspective, 720 min corresponds approximately to one school term of physical education in many educational systems (e.g., two 40–45 min lessons per week over 8–10 weeks). This indicates that the threshold identified in the analysis is not an exceptional intervention dosage but rather aligns with the duration of a sustained instructional unit or season-based curriculum.

Furthermore, instructional duration was associated with reductions in heterogeneity in selected comparisons in the global analysis. Notably, in the >1,440 min subgroup for decision-making, heterogeneity was completely eliminated (I^2^ = 0.0%), confirming that the apparent inconsistency in previous research is largely an artifact of inadequate implementation length rather than model incoherence.

Theoretically, this confirms a period of “pedagogical latency.” Deep learning—whether automatizing complex motor skills or internalizing tactical structures—cannot be rushed. Short, “unit-based” trials fail to allow the core social and psychological mechanisms (e.g., the peer trust in CL or the role identity in SE) to mature sufficiently to impact performance (Psotta and Martin, 2011; Nathan, 2016). Consequently, sufficient dosage (≥720 min) must be regarded not as a variable, but as a prerequisite for success.

Importantly, this finding highlights a logistical consideration for practitioners: short instructional blocks or fragmented units may be insufficient for pedagogical models to activate their intended social, cognitive, and motivational mechanisms. Thus, the “720-min rule” should be interpreted as a realistic planning benchmark rather than an experimental ideal.

### 4.4 From evidence to practice: a strategic, goal-oriented decision framework

Synthesizing these insights, we propose a practical decision framework to guide evidence-informed pedagogical selection. When applying this framework, educators should consider not only model selection but also whether sufficient instructional continuity can be scheduled to meet the minimum effective duration identified in this study. Without this temporal commitment, even theoretically well-aligned models may fail to produce meaningful learning gains.

Scenario A: The “Integrated Performance” Priority (Tactics + Skill). For comprehensive development in complex team sports, Hybrid Pedagogical Models (HPMs) may represent a comparatively favourable option. Our data confirms HPMs as the top-ranked model for both tactical decision-making (SUCRA = 80.1%) and skill execution (SUCRA = 77.2%). By integrating the cognitive scaffolding of TGfU with the situated pressure of SE, HPMs effectively bridge the gap between “knowing” and “doing.”

Scenario B: The “Intrinsic Motivation” Priority. To combat student disengagement or passivity, Teaching Games for Understanding (TGfU) should be prioritized. Ranking highest for motivation (SUCRA = 82.1%), TGfU’s cognitively engaging questioning strategies stimulate the learner’s desire to participate and solve problems, even before technical mastery is achieved.

Scenario C: The “Belonging & Enjoyment” Priority. To build a cohesive class culture and foster emotional attachment to sport, Sport Education (SE) is optimal (Enjoyment SUCRA = 74.0%). Teachers should commit to a full “season” structure to leverage its role-based affiliation, which creates an autonomy-supportive micro-society that sustains long-term enjoyment.

Scenario D: The “Technical Refinement” Priority. For focused, high-repetition refinement of specific motor actions (e.g., isolated drills), Cooperative Learning (CL) remains a powerful tool (Skill SUCRA = 75.5%). Implementing CL with structured peer-assessment protocols activates the “distributed scaffolding” mechanism necessary for correcting technical errors.

Scenario E: The “Social–Emotional Foundation” Priority. For groups requiring emotional regulation, initiate with TPSR to establish a safe, accountable environment before layering in other models.

Crucially, the “720-Minute Rule” applies universally. Rather than representing an intensified intervention, this duration reflects the minimum sustained exposure required for pedagogical mechanisms to stabilize within authentic school timetables.

## 5 Conclusion

This Network Meta-Analysis (NMA) of 65 studies provides an evidence-informed framework that may assist goal-oriented pedagogical decision-making for pedagogical selection in physical education. The findings indicate differentiated patterns of effectiveness across pedagogical models, challenging the traditional “one-size-fits-all” approach while emphasizing that observed differences should be interpreted as probabilistic tendencies rather than definitive hierarchies of superiority.

Comparative Trends Across Outcomes: Hybrid Pedagogical Models (HPMs) showed the highest probability rankings for tactical decision-making (SUCRA = 80.1%) and skill execution (SUCRA = 77.2%), suggesting their potential value in facilitating cognitive–motor integration. Teaching Games for Understanding (TGfU) ranked highest for promoting student motivation (SUCRA = 82.1%), likely due to its cognitively engaging, problem-based structure. Sport Education (SE) demonstrated favourable rankings for fostering enjoyment (SUCRA = 74.0%), reflecting its emphasis on sustained affiliation and social belonging. Cooperative Learning (CL) also showed comparatively strong rankings for skill execution (SUCRA = 75.5%).

However, league table analyses revealed substantial overlap in credible intervals among models, indicating that these rankings represent relative likelihoods of benefit rather than statistically definitive advantages.

Interpretation of Skill-Execution Findings: The significant publication bias detected for skill execution (Egger’s test *p* = 0.0077) suggests that the apparent strength of HPMs and CL in this domain may be influenced by underreporting of null findings. Accordingly, conclusions regarding skill-related benefits should be interpreted cautiously and viewed as context-dependent rather than universally generalizable.

The “Dosage” Effect: Instructional duration emerged as a key moderator of outcomes, with a dose–response relationship observed and approximately 720 min identified as a critical implementation threshold. Importantly, this duration corresponds roughly to one academic term of PE in many school systems (e.g., two 40–45 min lessons per week over 8–10 weeks), indicating that the threshold reflects a realistic curricular timespan rather than an intensified intervention requirement. This finding underscores that sustained pedagogical exposure—not merely model selection—is necessary for meaningful learning gains.

### 5.1 Practical implications

To support model-based instructional decision-making, PE teachers should adopt a goal-oriented selection framework guided by the principles of “outcome alignment” and “sufficient instructional duration”: (1) For Integrated Tactical and Skills Development: Hybrid Pedagogical Models may provide a productive structure for linking tactical understanding with applied performance, particularly in complex team sports. (2) For Enhancing Student Motivation: Teaching Games for Understanding can be used to stimulate engagement through problem-solving and contextual learning. (3) For Promoting Enjoyment and Belonging: Sport Education is well suited to longer instructional units that emphasize affiliation, identity, and shared responsibility. (4) For Focused Skill Refinement: Cooperative Learning structures can enhance feedback density and peer-supported correction during practice. (5) For Establishing Social–Emotional Foundations: TPSR can be used to cultivate responsibility and classroom climate prior to integrating other models.

Across all scenarios, adequate instructional continuity is essential. The findings suggest that pedagogical models require sustained implementation approaching a full instructional unit (≈720 min) for their mechanisms to take effect; fragmented or short-duration applications may fail to realize anticipated benefits. Rather than prescribing a universally “best” model, this framework supports adaptive alignment between pedagogical approach, intended learning outcome, and realistic school scheduling conditions.

### 5.2 Study limitations

Several limitations warrant acknowledgement. First, despite a multilingual search strategy (English, Chinese, and Spanish), the exclusion of studies in other languages may introduce language bias. Second, the predominance of quasi-experimental designs limits causal inference compared to randomized controlled trials, although it enhances ecological validity in school contexts. Third, a formal certainty appraisal system (e.g., GRADE) was not applied. Fourth, variations in implementation fidelity and teacher expertise were inconsistently reported and could not be modelled directly. Fourth, the review protocol was not prospectively registered in PROSPERO or another registry. Although the study followed PRISMA guidelines and applied predefined eligibility and analytical procedures, the absence of preregistration may increase the risk of unintentional procedural bias.

Fifth, regarding publication bias, Egger’s test indicated significant asymmetry for skill execution (*p* = 0.0077), suggesting that small studies with null or negative findings may be underrepresented. This limits confidence in the apparent strength of certain models for technical outcomes and reinforces the need for cautious interpretation of ranking results.

Finally, substantial heterogeneity was observed in several comparisons. While random-effects modelling and subgroup analyses addressed this statistically, the remaining variability likely reflects contextual diversity inherent in educational interventions rather than true inconsistency between pedagogical models. Notably, extended-duration interventions reduced heterogeneity, supporting the interpretation that implementation length is a critical explanatory factor.

## Data Availability

The original contributions presented in the study are included in the article/Supplementary material, further inquiries can be directed to the corresponding author.
