# Supplementary material for: A network meta-analysis of pedagogical models in physical education: evaluating multidimensional learning outcomes and instructional duration effects
Source: Front Psychol. 2026 Mar 3;17:1766890. doi: 10.3389/fpsyg.2026.1766890 (PMC12992282; doi:10.3389/fpsyg.2026.1766890)
Supplement: Supplementary file 1 [file Supplementary_file_1.docx]

A Network Meta-Analysis of Pedagogical Models in Physical Education: Evaluating Multidimensional Learning Outcomes and Instructional Duration Effects

Supplementary Material S1. Full Electronic Search Strategy

Database: Web of Science Core Collection

Search Date: 30 June 2025

Time Coverage: January 2000 to June 2025

Indexes: SCI-EXPANDED, SSCI, ESCI

Document Type: Article

Language: English, Spanish, Chinese

The search strategy was developed based on key pedagogical model terminology, physical education context terms, outcome variables, and intervention design keywords. The final search string used in Web of Science Core Collection was: TS = (("sport education" OR "cooperative learning" OR "teaching games for understanding" OR "tactical games" OR "game-based approach" OR "tactical games approach" OR "tactical approach" OR "tactical games model" OR "teaching for personal and social responsibility" OR "hybrid pedagogical model" OR "pedagogical model") AND ("sport" OR "physical education" OR "training") AND ("decision making" OR "skill execution" OR "motivation" OR "enjoyment") AND ("intervention" OR "experimental" OR "quasi-experimental" OR "randomized controlled trial")). The Web of Science Topic field (TS) includes: Title, Abstract, Author Keywords, Keywords Plus.

**Supplementary Table S1. Basic characteristics of the included studies.**

| Study/Nation | Sample/Age | Scope/Sport | Time | Minute (min) | | Intervention | Outcomes | Quality |
| --- | --- | --- | --- | --- | --- | --- | --- | --- |
| (Alcalá & Garijo, 2017), Spain | N=237 (58.3%male, 41.7females), EG=128, CG=109, Age=13.32±2.31 | Middle school (grade 7-10), basketball, cricket, handball | 24-lessons (55 min/ses) | 1320 | EG=TGfU. CG=TA | | ③ | MQ |
| (Altınkök, 2017), Iatanbul | N=68, EG=34, CG=34; Age=6-7, | Primary school (grade 1), basic motor skills | 12 weeks (1 ses/week, 120 min/ses) | 1440 | EG=CL  CG=TA | | ② | MQ |
| (Amado et al., 2014), Spain | N=47, EG=20, CG=27, Age=14.84±0.48 | Middle school (grade 4), dance | 12 weeks (2 ses/week, 50 min/ses) | 1200 | EG=SE  CG=TA | | ③ | LQ |
| (Andrianto, 2023), Indonesia | N=42, EG=21, CG=21 | College student (Physical education study); soccer | _ |  | EG=TGfU  CG=TA | | ③ | LQ |
| (Ben Khalifa et al., 2020), Tunisia | N=30 (Male=18; female=12), EG=15, CG=15, Age=15±0.4 | Middle school (grade 9); soccer | 12-lesson (60 min/ses) | 720 | EG=CL  CG=TA | | ①② | HQ |
| (Buendía et al., 2022), Spain | N=85 (male=46, female=39), EG=43, CG=42, Age=16.42±0.5 | High school, Ultimate frisbee | 5 weeks (2 ses/week, 55 min/ses) | 550 | EG=SE+TGfU  CG=TPSR | | ④ | MQ |
| (Cecchini et al., 2003), Spain | N=142, EG=72 (male=36, female=37), CG=70 (male=34, female=36), Age=12.7 | Middle school (grade 7), football hall | 10-lesson (60 min/ses) | 600 | EG=TPSR  CG=TA | | ④ | MQ |
| (Cecchini Estrada et al., 2019), Spain | N=372 (male=202; female=170), EG=182; CG=190, Age=12-17(14.2±2.34) | Middle schools (grade 4), Basketball, volleyball, dance | 6 months | - | EG=CL  CG=TA | | ③ | MQ |
| (Cecchini et al., 2007), Spain | N=186, EG=63, CG=61, male=92 (13.4±0.31), female=94 (13.8±0.30) | Middle school, soccer | 2 months (60 min/ses, 20 ses) | 1200 | EG=TPSR  CG=TA | | ④ | HQ |
| (Chang et al., 2016), Taiwan | N=126 (male=65, female=61), EG=61,CG=65, Age=13.7±0.5 | Primary school (grade 6), running, jumping, Chinese yo-yo, vaulting boxes, badminton, and basketball | 6 weeks (2 ses/week, 40 min/ses) | 480 | EG=SE  CG=TA | | ③ | HQ |
| (Chatzipanteli et al., 2015), Greece | N=601 (male=318, female=283), EG=316, CG=285, Age=13 | Middle school (grade 7), basketball, volleyball, soccer, track and field, Fitness enhancement, gymnastics | 16 weeks (3 ses/week, 45 min/ses) | 2160 | EG=SE  CG=TA | | ③④ | HQ |
| (Chatzopoulos et al., 2006), Greece | N=72(female), EG=35, CG=37, Age=12-13 | Middle schools (grade 7), soccer | 5 weeks (3 ses/week, 45 min/ses) | 675 | EG=TGfU  CG=TA | | ①②③ | HQ |
| (Chu et al., 2022), China | N=60, EG=30 (male=14, female=16), CG=30 (male=14, female=16) | College students, basketball | 8 weeks (2 ses/week, 45-50 min/ses) | 720-800 | EG=SE  CG=TA | | ③ | HQ |
| (Choi et al., 2021), USA | N=372, EG=184, CG=188, Age=18.5 | College students, various projects | 10 weeks (1 ses/week, 90 min/ses) | 900 | EG=SE  CG=TA | | ③④ | HQ |
| (Cuevas et al., 2015), Spain | N=86 (male=37, female=49), EG=43, CG=43, Age=15-17 | Middle school (grade 4), volleyball | 19 ses (55 min/ses) | 1045 | EG=SE  CG=TA | | ④ | MQ |
| (Cuevas et al., 2016), Spain | N=86 (male=37, female=49), EG=43, CG=43, Age=15-17 | Middle school (grade 4), volleyball | 19 ses (55 min/ses) | 1045 | EG=SE  CG=TA | | ③④ | MQ |
| (Darnis & Lafont, 2015), France | N=52(female), EG=26, CG=26, Age=11.1±0.36 | Primary school (grades 3-4), basketball and handball | 10weeks (1 ses/week, 45 min/ses) | 450 | EG=CL+TGFU CG=TA | | ② | MQ |
| (Ebrahimi & Hasan, 2025), Iran | N=32, EG=16, CG=16, Age=13-14 | Middle school (grade 7), volleyball | 12 weeks (2 ses/week, 70 min/ses) | 1680 | EG=SGTSP+SE  CG=SGTSP | | ①② | MQ |
| (Fernández-Río et al., 2017), Spain | N=217 (male=113, female=104), EG=217, CG=217, Age=12-17 | High school (grade 8-11), ultimate-frisbe | 6 weeks (2 ses/week, 55 min/ses) | 660 | EG=SE  CG=TA | | ③④ | HQ |
| (Fernandez-Rio et al., 2017), Spain | N=249, EG=137 (male=66, female=71), CG=112 (male=56, female=56), Age=EG(13.91±1.76), CG(13.41±1.25) | Middle school, fitness training | 16 weeks (2 ses/week, 120 min/ses) | 3840 | EG=CL  CG=TA | | ③ | HQ |
| (Fernández-Río et al., 2014), Spain | N=264 (male=88, female=176), EG=130, CG=134, Age=18-42(female=19.97, male=20.81) | College students, mixed sports | 12 weeks (2 ses/week, 60 min/ses) | 1440 | EG=CL  CG=TA | | ③④ | MQ |
| (García-Castejón et al., 2021), Spain | N=99, EG=44, CG=55,  Age=12.63±0.72 | Middle school, basketball, futsal, volleyball | 11 weeks (1 ses/week, 40-45 min/ses) | 440-495 | EG=TPSR+TGfU  CG=TA | | ③④ | HQ |
| (Gil et al., 2019), Spain | N=37, EG=17, CG=20, Age=11.22±0.422 | Primary school (grade 6), basketball | 18weeks (1 ses/week, 45 min/ses) | 810 | EG=TGfU  CG=TA | | ①② | HQ |
| (Gil-Arias et al., 2017), Spain | N=55, EG=27, CG=28, Age=11.22±0.422 | Middle school, handball, basketball, hockey | 8 weeks (2 ses/week, 45-60 min/ses) | 720-960 | EG=TGfU+SE  CG=TA | | ③④ | HQ |
| (Gil-Arias et al., 2021), Spain | N=292, EG=148 (male=77, female=71),CG=144 (male=75, female=69), Age=10.41±0.49 | Primary school, basketball | 8weeks (2 ses/week, 50 min/ses) | 800 | EG=TGfU+SE  CG=TA | | ③④ | MQ |
| (Gray & Sproule, 2011), UK | N=52 (male=28, female=24), EG=27,CG=25, Age=12.5±0.3 | Middle school, basketball | 5 weeks (1 ses/week, 80 min/ses) | 400 | EG=TGfU  CG=TA | | ①② | HQ |
| (Guijarro-Romero et al., 2018), Spain | N=85, EG (low level) = 23; CG=42, Age=10-12 | Primary school (grade 5-6), soccer and basketball | 8 weeks (2 ses/week, 40 min/ses) | 640 | EG=TGfU  CG=TA | | ①② | MQ |
| (Hastie et al., 2013), USA | N=68, EG=30, CG=26, Age=16years, 4months | High school (grade 10), track and field | 10-lessons (90 min/ses) | 900 | EG=SE  CG=TA | | ② | MQ |
| (Hernández-Andreo et al., 2020), Spain | N=93, EG=52 (male=31, female=21), CG=41 (male=23,female=18), Age=13.32±0.62 | Middle school (grade 7), alternative sports | 6 weeks (2 ses/week, 50 min/ses) | 600 | EG=SE  CG=TA | | ③ | HQ |
| (Lemus et al., 2016), Spain | N=46, EG=22, CG=24, Age=14-15 | High school, basketball | 4.5 weeks (2 ses/week, 45 min/ses) | 405 | EG=TGfU  CG=TA | | ①② | MQ |
| (Luo et al., 2020), Taiwan | N=108 (male=86, female=22), EG=52, CG=56, Age=22.09±0.64 | College physical education, basketball | 12 weeks (1 ses/week, 100 min/ses) | 1200 | EG=TGT(Team-Game-Tournaments),(TGfU) CG=TA | | ②③ | HQ |
| (Manzano-Sánchez, 2023), Spain | N=120, EG=61 (male=27, female=34), CG=59 (male=32, female=27), Age=13.48±1.36 | Middle school, fitness training | 4 weeks (2 ses/week, 55 min/ses) | 440 | EG=GF+CL  CG=TA | | ④ | MQ |
| (Manzano-Sánchez et al., 2019), Spain | N=85, EG=35 (male=18, female=17), CG=50 (male=22, female=28), Age=16.22±0.41 | High schools, physical education subject | 8 months | - | EG=TPSR  CG=TA | | ③④ | MQ |
| (Manzano-Sánchez & Valero-Valenzuela, 2019), Spain | N=272, EG=227, CG=45, Age=11.13±1.78 | Primary school, physical education subject | 7 months (2-3 ses/week, 45 min/ses | 2700-4050 | EG=TPSR  CG=TA | | ③ | HQ |
| (Manzano-Sánchez et al., 2021), Spain | N=167, EG=100 (male=37, female=63), CG=67 (male=37, female=30), Age=15.28±3.20 | Middle school, physical education | 8 months (2 ses/week, 45 min/ses) | 3060 | EG=TPSR  CG=TA | | ③ | MQ |
| (Manzano-Sánchez & Gómez-López, 2023), Spain | N=408, EG=216, CG=192, Age=10-14 | Primary school, physical education | 5 months ( ＞60% lessons) | - | EG=TPSR  CG=TA | | ③ | HQ |
| (Medina-Casaubón & Burgueño, 2017), Spain | N=44 (male=22,female=22), EG=22, CG=22, Age=16.32±0.57 | High school, basketball | 6 weeks (2 ses/week, 55 min/ses) | 660 | EG=SE  CG=TA | | ③ | HQ |
| (Melero et al., 2021), Spain | N=69; EG=26 (male=14, female=12) CG=32 (male=15, female=17), Age=13-15 | Middle schools; motivation, physical fitness, lifestyle habits | 9 months (4 ses/week, 60 min/ses) | 9360 | EG=  TPSR+GF  CG=TA | | ③ | HQ |
| (Menickelli & Hastie, 2014), USA | N=40 (male=30, female=10), EG=20, CG=20, Age=15.9±1.1 | High school, Disc Lacrosse | 5 weeks (5 ses/week  70 min/ses) | 1750 | EG=SE  CG=TA | | ④ | HQ |
| (Merino-Barrero et al., 2019), Spain | N=72, EG=35 (male=21, female=14), CG=37 (male=17, female=20), Age=12.05±1.12 | Middle school (grade 7-8), indoor hockey, indoor soccer, basketball, traditional games | 29-lessons (45 min/ses) | 1305 | EG=TPSR  CG=TA | | ③ | HQ |
| (Miller et al., 2016), Australia | N=106, EG=54 (male==26, female==28), CG=52 (male==33, female=19), Age=10.7(9-12) | Primary school (grade 3-6), throw, catch, target games, net/wall games, invasion games | 6weeks (1 ses/week, 60 min/ses) | 360 | EG=GCA (TGfU)  CG=TA | | ①②④ | HQ |
| (Morales-Belando & Arias-Estero, 2017), Spain | N=67(male=45, female=22); EG=40, CG=27, Age=9.32±2.60 | A sailing primary school in South of Europe, Sailing | 2 months  (80 min/ses, 6 ses/week) | 960 | EG=TRfU (TGfU)  CG=TA | | ①②④ | LQ |
| (Nathan & Haynes, 2013), Malaysia | N=72(male), EG=36, CG=36, Age=±13 years | Middle schools, field hockey | 15 weeks (1 ses/week, 40 min/ses) | 600 | EG=TGfU  CG=TA | | ①② | MQ |
| (Nathan, 2016), Malaysia | N=32 (male=16, female=16), EG=16; CG=16, Age15.5±1.0 | Sport club, badminton | 8 weeks (2 ses/week, 45 min/ses) | 720 | EG=TGfU  CG=TA | | ①② | HQ |
| (Navarro-Patón et al., 2017), Spain | N=104 (male=59, female=45), EG=54, CG=50, Age=10.29±0.62 | primary school (grade 5-6), cooperative games | 3 weeks (2 ses/week,  45-60 min/week) | 270-360 | EG=CL+game  CG=TA | | ③④ | HQ |
| (Osman, 2017), Egypt | N=45, EG=24(20±1.9); CG=21(20±1.2) | College student, football | 10weeks | - | EG=TGfU  CG=TA | | ①② | MQ |
| (Pan et al., 2019), Taiwan | N=130, EG=75 (male=38, female=37), CG=58 (male=30,female=28), Age=EG=16.78±0.57, CG=16.82±0.57 | High school, volleyball | 16 weeks (2 ses/week,  45 min/ses) | 1440 | EG=TPSR+SE  CG=TPSR+TA | | ① | HQ |
| (Pan et al., 2023)，Taiwan | N=90, EG=46 (male=24, female=22), CG=44 (male=23, female=21), Age= EG=15.02±0.73, CG=14.78±0.73 | Middle school, basketball | 10 weeks (2 ses/week, 45 min/ses) | 900 | EG=TGfU+SE  CG=TGfU | | ①②③④ | HQ |
| (Pereira et al., 2016), Portugal | N=47 (male=25, female=22), EG=19, CG=28, Age=10.9±0.8 | Primary school, Track and field, shot put, triple jump and hurdles | 20-lessons (2-3 ses/week, 45 min/ses) | 1800-2700 | EG=SE  CG=TA | | ② | HQ |
| (Perlman, 2010), USA | N=78, EG=40 (male=14, female=26), CG=38 (male=10, female=28) | Middle school (grade 9-12), basketball, volleyball, soccer and lacrosse | 2 years (3-4 ses/week, 60 min/ses) | 18720 | EG=SE  CG=TA | | ④ | MQ |
| (Perlman, 2011), USA | N=182, EG=94 (male=43, female=51), CG=88 (male=48, female=40) | Middle school (grade 9), volleyball | 1 month (4 ses/week, 60 min/ses) | 960 | EG=SE  CG=TA | | ③ | MQ |
| (Perlman, 2012), Australia | N=50, EG=25 (male=16, female=9), CG=25 (male=18, female=7) | College (Pre-service physical education teachers), variety of team sports | 15 weeks (1 ses/week, 60 min/ses) | 900 | EG=SE  CG=TA | | ③ | HQ |
| (Práxedes et al., 2016), Spain | N=18, EG=9, CG=9; Age=10.7±0.6 | Club (experience in Spanish youth football league of 3-6 years), soccer | 12 weeks (2 ses/week, 60 min/ses) | 1440 | EG=TGfU  CG=TA | | ①② | HQ |
| (Práxedes et al., 2018), Spain | N=19 (male), EG=10, CG=9; EG=10.55±0.51, CG=11.77±0.66 | Club (Experience 4.88±1.05), soccer | 7 weeks (2 ses/week, 60 min/ses) | 840 | EG=NLP(TGfU)  CG=TA | | ①② | HQ |
| (Pritchard et al., 2008), USA | N=47, EG=26, CG=21 | Middle school (grade 9); volleyball | 20-lesson (5 ses/week  50 min/ses) | 1000 | EG=SE  CG=TA | | ①② | MQ |
| (Psotta & Martin, 2011), Czech Republic | N=24 (female), EG=12, CG=12,  Age=20.9±0.7 | College student (No any team sport experience), team sport | 5 weeks (2 ses/week, 85/25 min/ses) | 550 | EG=CTA (TGfU)  CG=TA | | ①② | HQ |
| (Sánchez et al., 2021), Spain | N=167, EG=100, CG=67, Age=15.97±2.31 | Middle schools, basketball, volleyball, handball | 8 months (2 ses/week) | - | EG=TPSR  CG=TA | | ③ | HQ |
| (Shariati et al., 2024), Iran | N=60, EG=30, CG=30, Age=13.32±1.65 | Middle school (grade 7), Table tennis | 24 weeks (2 ses/week, 50 min/ses) | 2400 | EG=TGfU+TPSR  CG=TA | | ② | HQ |
| (Sierra-Ríos et al., 2020), Spain | N=30 (male), EG=15, CG=15, Age=under 12 year | Club (experience of soccer 6.9±1.08 years), soccer | 6 weeks (2 ses/week, 80 min/ses) | 960 | EG=TGfU  CG=TA | | ② | HQ |
| (Spittle & Byrne, 2009), Australia | N=115 (male=97, female=18), EG=41, CG=74, Age=13-14 | Middle school (grade 8), soccer, hockey | 10 weeks (1 ses/week, 100 min/ses) | 1000 | EG=SE  CG=TA | | ③ | MQ |
| (Viciana et al., 2020), Spain | N=123 (male=60, female=63); EG=67; CG=42, Age=14-15 | Middle school (grade 9), volleyball small-sided games | 12-lessons (2-3 ses/week, 45-60 min/ses) | 1080-2160 | EG=SE  CG=TA | | ③④ | MQ |
| (Wallhead & Ntoumanis, 2004), UK | N=51, EG=26, CG=25, Age=14.5±0.48 | High school, basketball | 8 weeks (1 ses/week, 30-40 min/ses) | 240-320 | EG=SE  CG=TA | | ③ | LQ |
| (Wallhead et al., 2014), USA | N=568 (male=258, female=310), EG=261, CG=277, Age=14.75±0.48 | High school (grade 10); require physical education program | 2 years (2-3 ses/week, 45 min/ses) | 9154-14078 | EG=SE  CG=TA | | ④ | MQ |
| (Chenchen et al., 2019), China | N=64, EG=36, CG=28, Age=16-17 | High school, Table tennis | 16-lessons (1 ses/week, 40min/ses) | 640 | EG=SE  CG=TA | | ② | HQ |
| (Yang & Lu, 2013), China | N=100 (male), EG=50, CG=50 | College (University Sports institute 2011 level), football | 40 hours | 2400 | EG=TGfU  CG=TA | | ② | MQ |

N=sample size; EG=experimental group; CG= Control group; TA= Technical approach; ①=decision making; ②=skill execution③=motivation（including intrinsic motivation and autonomous motivation）; ④=enjoyment（including enjoyment and satisfaction）; SE= Sport Education; TGfU= teaching games for understanding（this paper classifies all game-centered methods into TGfU; including nonlinear pedagogy, NLP(Práxedes et al., 2018), teaching races for understanding, TRfU(Morales-Belando & Arias-Estero, 2017), combined tactical approach, CTA(Psotta & Martin, 2011), game-centered approach, GCA(Miller et al., 2016), ; CL= Cooperative Learning; TPSR= teaching for personal and social responsibility; HPM= Hybrid Pedagogical Model(including SE+TGfU, CL+TGfU, TPSR+TGfU, CL+game, GF+CL); LQ: Low quality; MQ: Medium quality; HQ: High quality.

**Supplementary Table S2. Result of the methodological quality evaluation.**

| First author | 1 | 2 | 3 | 4 | 5 | 6 | 7 | 8 | 9 | 10 | 11 | 12 | scores |
| --- | --- | --- | --- | --- | --- | --- | --- | --- | --- | --- | --- | --- | --- |
| (Alcalá & Garijo, 2017) | 2 | 1 | 2 | 2 | 1 | 2 | 0 | 0 | 1 | 2 | 1 | 2 | 16 |
| (Altınkök, 2017) | 2 | 2 | 2 | 2 | 0 | 2 | 1 | 0 | 2 | 2 | 1 | 2 | 18 |
| (Amado et al., 2014) | 2 | 1 | 2 | 2 | 0 | 1 | 1 | 0 | 1 | 2 | 1 | 2 | 15 |
| (Andrianto, 2023) | 2 | 1 | 2 | 2 | 0 | 1 | 0 | 0 | 1 | 2 | 0 | 2 | 13 |
| (Ben Khalifa et al., 2020) | 2 | 2 | 2 | 2 | 1 | 2 | 2 | 0 | 2 | 2 | 2 | 2 | 21 |
| (Buendía et al., 2022) | 2 | 1 | 2 | 2 | 1 | 1 | 0 | 0 | 2 | 2 | 1 | 2 | 16 |
| (Cecchini et al., 2003) | 2 | 1 | 2 | 2 | 1 | 1 | 0 | 0 | 2 | 2 | 2 | 2 | 17 |
| (Cecchini Estrada et al., 2019) | 2 | 1 | 2 | 2 | 0 | 2 | 1 | 0 | 2 | 2 | 2 | 2 | 18 |
| (Cecchini et al., 2007) | 2 | 2 | 2 | 2 | 0 | 2 | 2 | 0 | 2 | 2 | 1 | 2 | 19 |
| (Chang et al., 2016) | 2 | 2 | 2 | 2 | 0 | 1 | 2 | 0 | 2 | 2 | 2 | 2 | 19 |
| (Chatzipanteli et al., 2015) | 2 | 2 | 2 | 2 | 0 | 2 | 2 | 0 | 1 | 2 | 2 | 2 | 19 |
| (Chatzopoulos et al., 2006) | 2 | 2 | 2 | 2 | 1 | 2 | 2 | 0 | 2 | 2 | 2 | 2 | 21 |
| (Chu et al., 2022) | 2 | 2 | 2 | 2 | 0 | 1 | 2 | 0 | 2 | 2 | 2 | 2 | 19 |
| (Choi et al., 2021) | 2 | 2 | 2 | 2 | 1 | 2 | 1 | 2 | 2 | 2 | 2 | 2 | 22 |
| (Cuevas et al., 2015) | 2 | 2 | 2 | 2 | 0 | 1 | 2 | 0 | 2 | 2 | 1 | 2 | 16 |
| (Cuevas et al., 2016) | 2 | 2 | 2 | 2 | 0 | 1 | 2 | 0 | 2 | 2 | 1 | 2 | 18 |
| (Darnis & Lafont, 2015) | 2 | 1 | 2 | 2 | 1 | 1 | 0 | 0 | 2 | 2 | 2 | 1 | 16 |
| (Ebrahimi & Hasan, 2025) | 2 | 1 | 2 | 2 | 2 | 2 | 1 | 0 | 2 | 2 | 1 | 2 | 17 |
| (Fernández-Río et al., 2017) | 2 | 2 | 2 | 2 | 1 | 2 | 2 | 0 | 2 | 2 | 1 | 2 | 20 |
| (Fernandez-Rio et al., 2017) | 2 | 2 | 2 | 2 | 1 | 2 | 1 | 0 | 2 | 2 | 2 | 2 | 20 |
| (Fernández-Río et al., 2014) | 2 | 1 | 2 | 2 | 0 | 2 | 1 | 0 | 2 | 2 | 1 | 2 | 18 |
| (García-Castejón et al., 2021) | 2 | 2 | 2 | 2 | 0 | 2 | 1 | 0 | 2 | 2 | 2 | 2 | 19 |
| (Gil et al., 2019) | 2 | 1 | 2 | 2 | 2 | 2 | 2 | 0 | 2 | 2 | 1 | 2 | 20 |
| (Gil-Arias et al., 2017) | 2 | 2 | 2 | 2 | 0 | 2 | 2 | 0 | 2 | 2 | 1 | 2 | 19 |
| (Gil-Arias et al., 2021) | 2 | 2 | 2 | 2 | 0 | 1 | 0 | 0 | 2 | 2 | 1 | 2 | 16 |
| (Gray & Sproule, 2011) | 2 | 2 | 2 | 2 | 1 | 1 | 2 | 0 | 2 | 2 | 2 | 1 | 19 |
| (Guijarro-Romero et al., 2018) | 2 | 1 | 2 | 2 | 0 | 1 | 2 | 0 | 2 | 2 | 1 | 2 | 17 |
| (Hastie et al., 2013) | 2 | 1 | 2 | 2 | 1 | 2 | 2 | 0 | 1 | 2 | 1 | 2 | 18 |
| (Hernández-Andreo et al., 2020) | 2 | 1 | 2 | 2 | 2 | 2 | 2 | 1 | 2 | 2 | 2 | 2 | 22 |
| (Lemus et al., 2016) | 2 | 1 | 2 | 2 | 1 | 1 | 1 | 0 | 2 | 2 | 1 | 1 | 17 |
| (Luo et al., 2020) | 2 | 1 | 2 | 2 | 2 | 2 | 2 | 0 | 2 | 2 | 2 | 2 | 21 |
| (Manzano-Sánchez, 2023) | 2 | 1 | 2 | 2 | 0 | 1 | 1 | 0 | 9 | 2 | 1 | 2 | 16 |
| (Manzano-Sánchez et al., 2019) | 2 | 1 | 2 | 2 | 0 | 2 | 0 | 0 | 1 | 2 | 1 | 2 | 15 |
| (Manzano-Sánchez & Valero-Valenzuela, 2019) | 2 | 1 | 2 | 2 | 1 | 2 | 2 | 0 | 1 | 2 | 2 | 2 | 19 |
| (Manzano-Sánchez et al., 2021) | 2 | 1 | 2 | 2 | 1 | 2 | 2 | 0 | 1 | 2 | 1 | 2 | 18 |
| (Manzano-Sánchez & Gómez-López, 2023) | 2 | 1 | 2 | 2 | 1 | 2 | 2 | 0 | 2 | 2 | 2 | 2 | 20 |
| (Medina-Casaubón & Burgueño, 2017) | 2 | 1 | 2 | 2 | 1 | 2 | 2 | 0 | 2 | 2 | 1 | 2 | 19 |
| (Melero et al., 2021) | 2 | 2 | 2 | 2 | 0 | 2 | 1 | 0 | 2 | 2 | 2 | 2 | 19 |
| (Menickelli & Hastie, 2014) | 2 | 2 | 2 | 2 | 0 | 1 | 2 | 0 | 2 | 2 | 2 | 2 | 19 |
| (Merino-Barrero et al., 2019) | 2 | 1 | 2 | 2 | 1 | 2 | 2 | 0 | 2 | 2 | 2 | 2 | 20 |
| (Miller et al., 2016) | 2 | 1 | 2 | 2 | 2 | 2 | 2 | 0 | 2 | 2 | 2 | 2 | 21 |
| (Morales-Belando & Arias-Estero, 2017) | 2 | 1 | 2 | 2 | 0 | 2 | 0 | 0 | 2 | 2 | 1 | 2 | 15 |
| (Nathan & Haynes, 2013) | 2 | 1 | 2 | 2 | 0 | 2 | 1 | 0 | 2 | 2 | 1 | 2 | 17 |
| (Nathan, 2016) | 2 | 2 | 2 | 2 | 1 | 1 | 2 | 0 | 2 | 2 | 2 | 1 | 21 |
| (Navarro-Patón et al., 2017) | 2 | 1 | 2 | 2 | 1 | 2 | 2 | 0 | 2 | 2 | 2 | 2 | 20 |
| (Osman, 2017) | 2 | 1 | 2 | 2 | 0 | 2 | 0 | 0 | 2 | 2 | 2 | 2 | 17 |
| (Pan et al., 2019) | 2 | 1 | 2 | 2 | 1 | 2 | 2 | 0 | 2 | 2 | 2 | 2 | 20 |
| (Pan et al., 2023) | 2 | 2 | 2 | 2 | 1 | 2 | 2 | 0 | 2 | 2 | 1 | 2 | 20 |
| (Pereira et al., 2016) | 2 | 1 | 2 | 2 | 2 | 2 | 2 | 0 | 2 | 2 | 2 | 2 | 21 |
| (Perlman, 2010) | 2 | 1 | 2 | 2 | 0 | 1 | 2 | 0 | 2 | 2 | 2 | 2 | 18 |
| (Perlman, 2011) | 2 | 1 | 2 | 2 | 0 | 1 | 1 | 0 | 2 | 2 | 1 | 2 | 16 |
| (Perlman, 2012) | 2 | 2 | 2 | 2 | 1 | 1 | 2 | 0 | 2 | 2 | 2 | 2 | 20 |
| (Práxedes et al., 2016) | 2 | 2 | 2 | 2 | 1 | 2 | 2 | 0 | 2 | 2 | 2 | 2 | 21 |
| (Práxedes et al., 2018) | 2 | 2 | 2 | 2 | 1 | 2 | 2 | 0 | 2 | 2 | 2 | 2 | 21 |
| (Pritchard et al., 2008) | 2 | 1 | 2 | 2 | 1 | 2 | 0 | 0 | 2 | 2 | 1 | 2 | 17 |
| (Psotta & Martin, 2011) | 2 | 2 | 2 | 2 | 1 | 1 | 1 | 0 | 2 | 2 | 2 | 2 | 21 |
| (Sánchez et al., 2021) | 2 | 1 | 2 | 2 | 1 | 2 | 2 | 0 | 2 | 2 | 2 | 2 | 20 |
| (Shariati et al., 2024) | 2 | 1 | 2 | 2 | 1 | 2 | 2 | 0 | 2 | 2 | 2 | 2 | 20 |
| (Sierra-Ríos et al., 2020) | 2 | 2 | 2 | 2 | 0 | 1 | 2 | 1 | 2 | 2 | 2 | 2 | 20 |
| (Spittle & Byrne, 2009) | 2 | 1 | 2 | 2 | 0 | 2 | 1 | 0 | 1 | 2 | 1 | 2 | 16 |
| (Viciana et al., 2020) | 2 | 2 | 2 | 2 | 0 | 1 | 0 | 0 | 2 | 2 | 2 | 2 | 17 |
| (Wallhead & Ntoumanis, 2004) | 2 | 1 | 1 | 2 | 0 | 1 | 0 | 0 | 1 | 2 | 2 | 1 | 13 |
| (Wallhead et al., 2014) | 2 | 1 | 2 | 2 | 0 | 2 | 0 | 0 | 2 | 2 | 1 | 2 | 16 |
| (Chenchen et al., 2019) | 2 | 1 | 1 | 2 | 1 | 2 | 2 | 0 | 2 | 2 | 2 | 2 | 19 |
| (Yang & Lu, 2013) | 2 | 1 | 2 | 2 | 0 | 2 | 0 | 0 | 2 | 2 | 2 | 2 | 17 |

Table Note: “1” indicates that the purpose of the study was clearly given; “2” indicates the consistency of the patients

included; “3” indicates the expected data collection; “4” indicates whether the outcome indicators reflected the

purpose of the study; “5” indicates whether the trial was blinded; “6” indicates whether the follow-up period was

adequate; “7” indicates whether the loss of follow-up rate was less than 5%; “8” indicates whether the sample size

was estimated; “9” indicates whether the selection of the control group was appropriate; “10” indicates whether the

control groups indicated whether the control groups are synchronised; “11” indicates whether the baselines were

comparable between groups; “12” indicates whether the statistical analysis was appropriate.

**Supplementary Figure S1. Trace plots and posterior density plots for assessing convergence of Bayesian network meta-analysis model.**

| **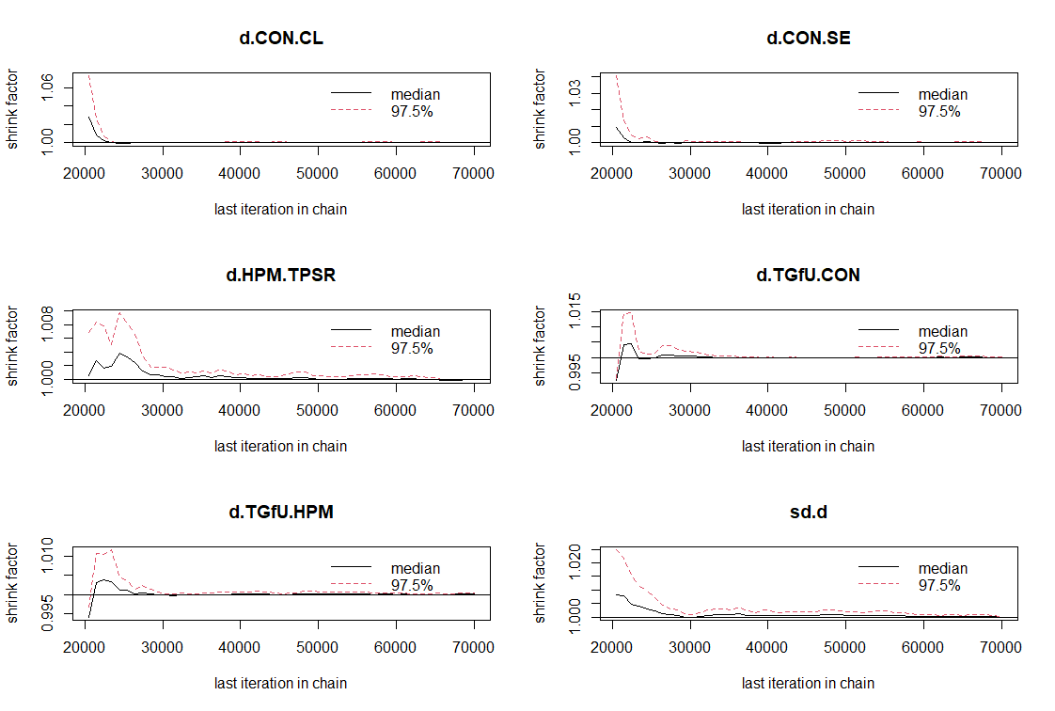** | **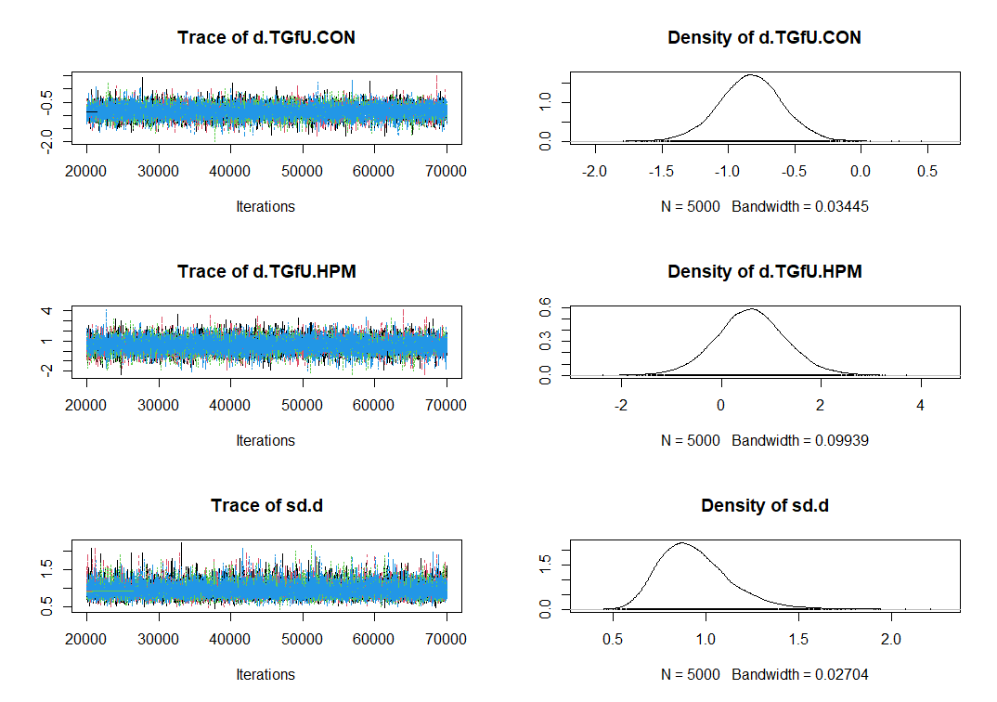** |
| --- | --- |

**(A) Decision making outcome: trace plots (left) and posterior density plots (right)**

| **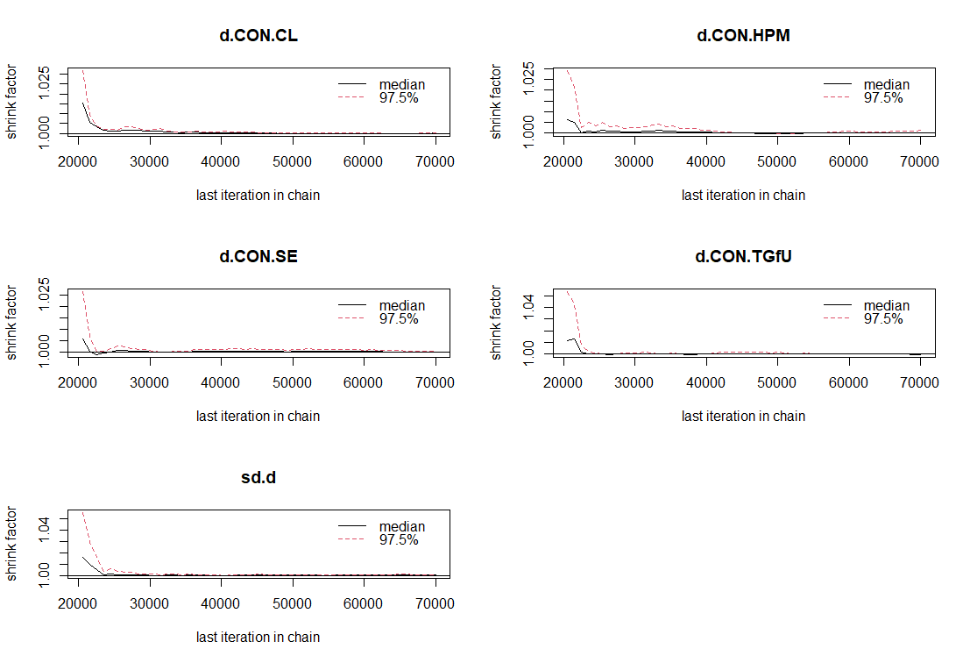** | **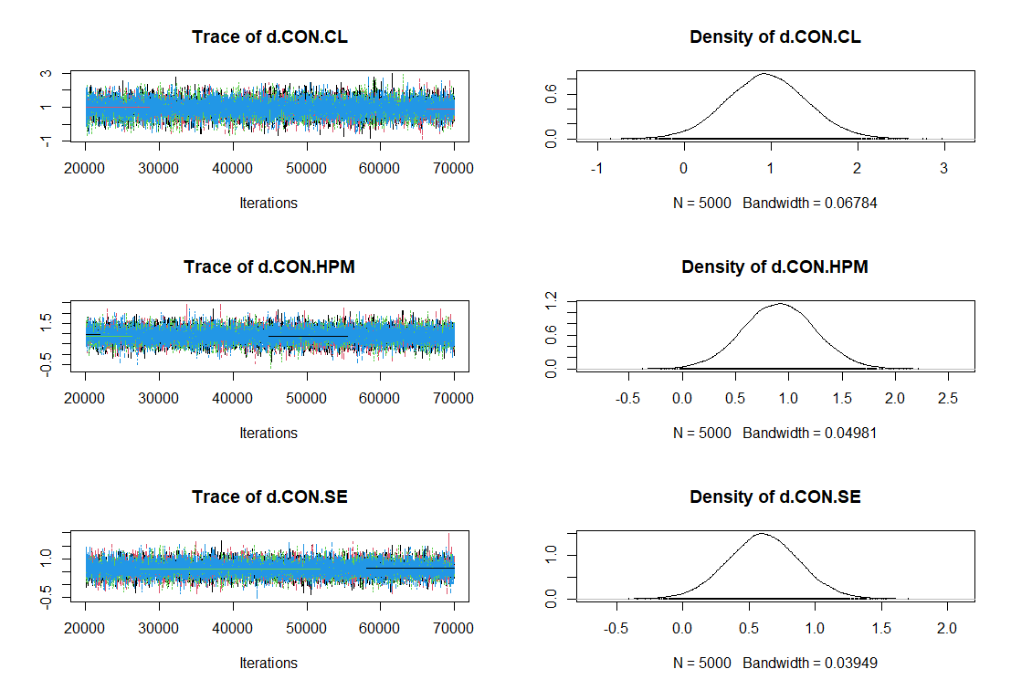**  **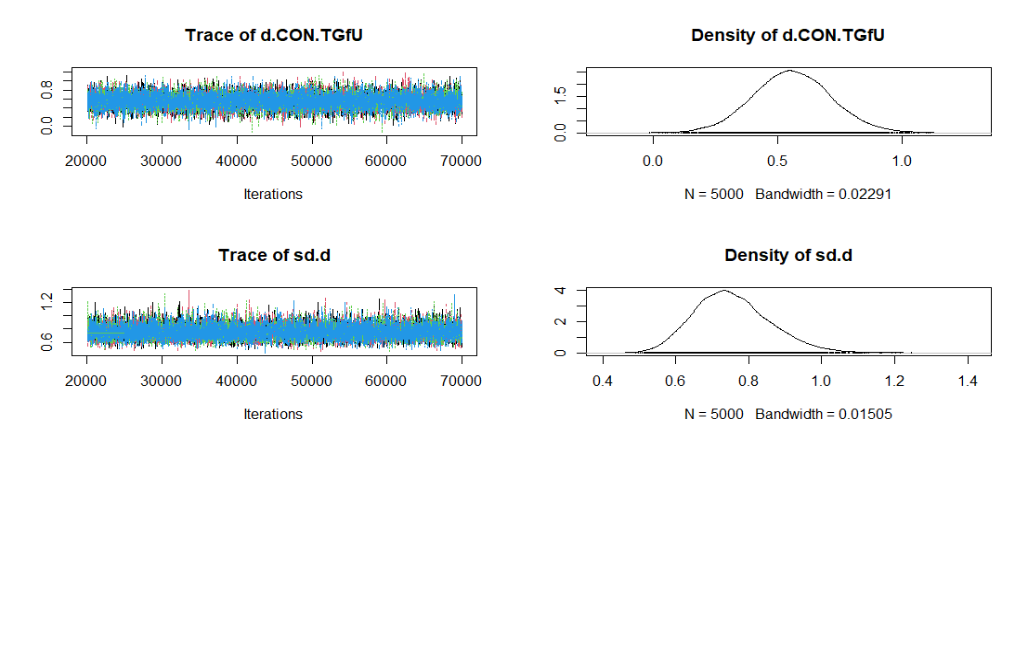** |
| --- | --- |

**(B) Skill execution outcome: trace plots (left) and posterior density plots (right).**

| **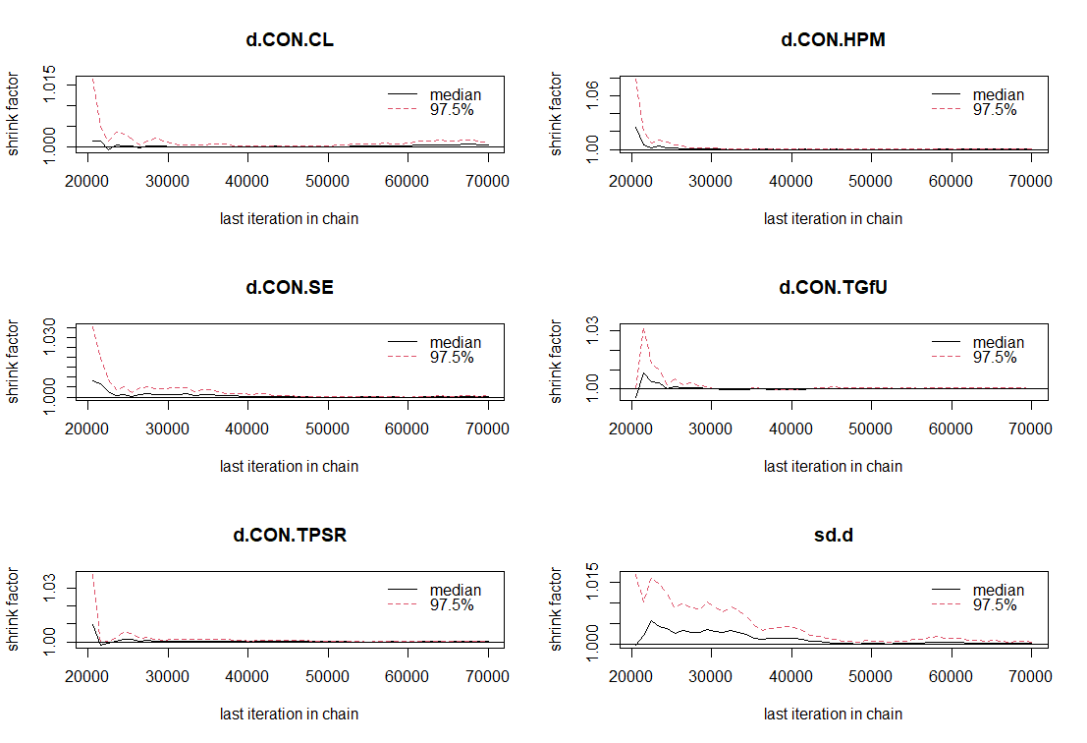** | **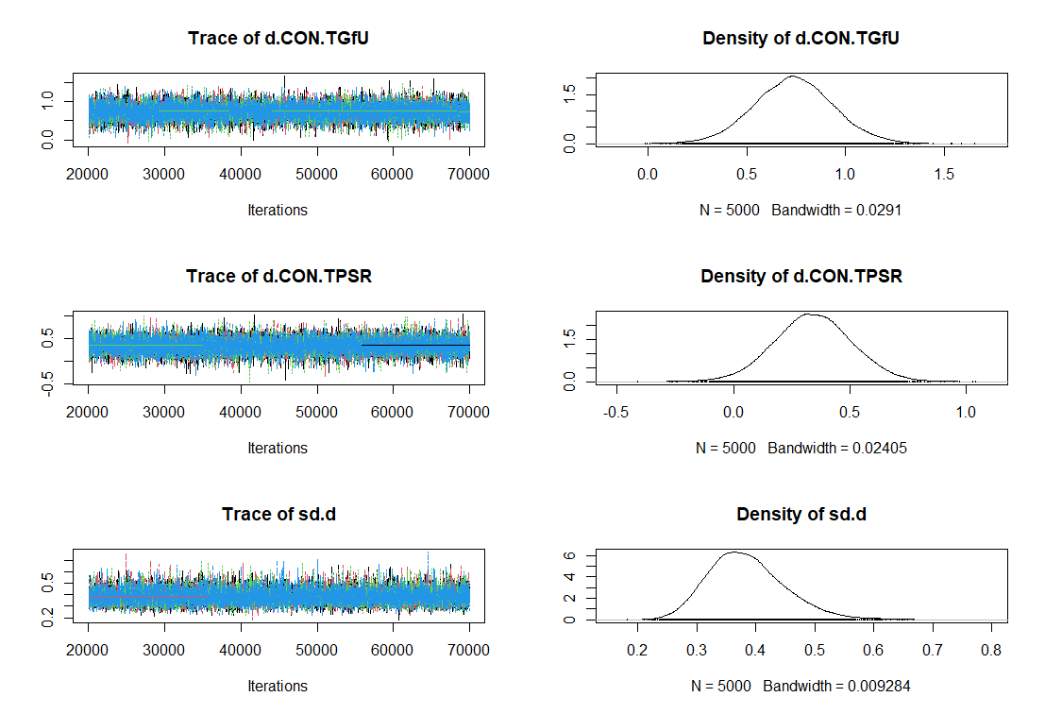** |
| --- | --- |

**(C) Motivation outcome: trace plots (left) and posterior density plots (right).**

| **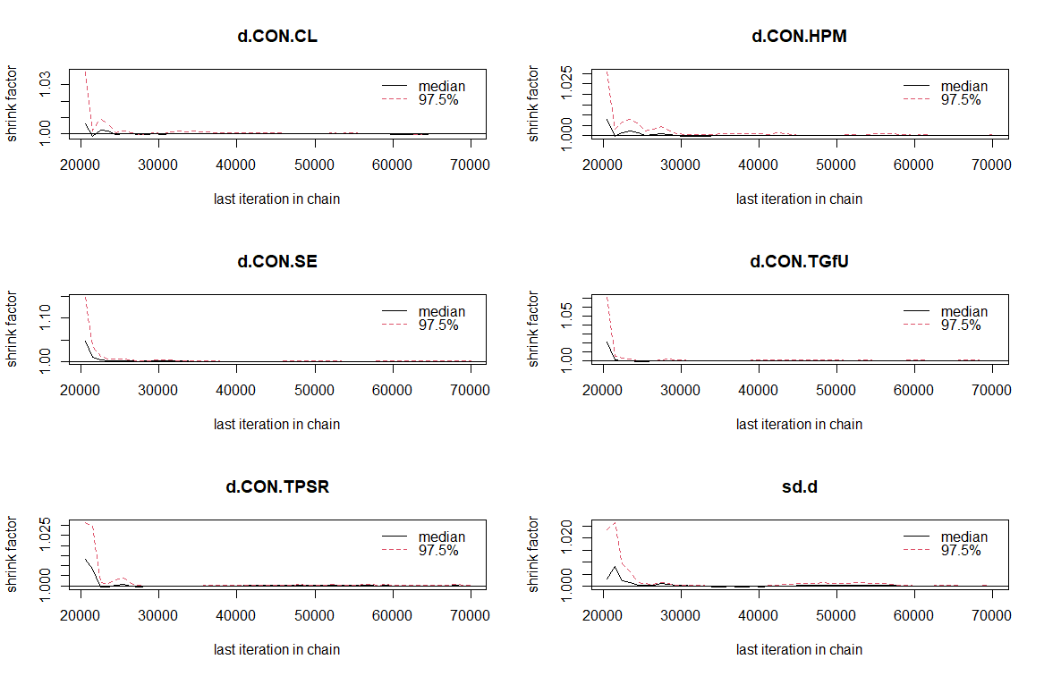** | **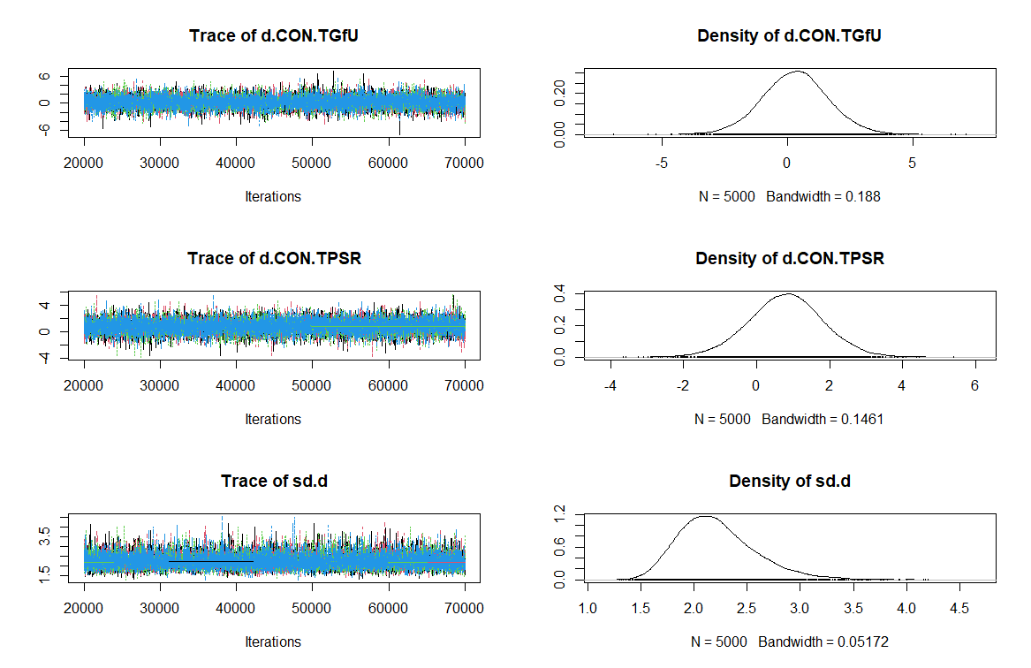** |
| --- | --- |

**(D) Enjoyment outcome: trace plots (left) and posterior density plots (right).**

**Supplementary Table S3. Different indicators node splitting method test.**

|  | Intervention methods | Direct MD（95%CI） | Indirect MD (95%CI) | Network MD (95%CI) | P |
| --- | --- | --- | --- | --- | --- |
| Skill execution | CON vs. TGfU | 0.59(0.27,0.91) | 0.071(-1.2,1.4) | 0.56(0.25,0.87) | 0.445 |
|  | CON vs. HPM | 0.67(-0.26,1.6) | 1.2(0.19,2.2) | 0.91(0.23,1.6) | 0.446 |
|  | TGfU vs HPM | 0.60(-0.33,1.5) | 0.085(-0.90,1.1) | 0.35(-0.32,1.0) | 0.448 |
| motivation | CON vs. TGfU | 0.89(0.45,1.3) | 0.055(-0.86,0.96) | 0.73(0.34,1.1) | 0.103 |
|  | CON vs. HPM | 0.50(0.17,0.82) | 1.3(0.37,2.3) | 0.58(0.26,0.89) | 0.101 |
|  | TGfU vs HPM | 0.44(-0.41,1.3) | -0.39(-0.94,0.15) | -0.15(-0.62,0.31) | 0.103 |
| enjoyment | CON vs. TGfU | -0.075(-3.3,3.2) | 1.2(-3.7,6.0) | 0.30(-2.3,2.9) | 0.664 |
|  | CON vs. TPSR | 0.68(-1.6,3.0) | 1.3(-3.5,6.2) | 0.80(-1.2,2.8) | 0.801 |
|  | CON vs. HPM | 1.5(-0.23,3.2) | 0.47(-3.3,4.2) | 1.3(-0.22,2.8) | 0.614 |
|  | TGfU vs HPM | 0.26(-4.3,4.8) | 1.5(-2.2,5.1) | 1.0(-1.8,3.8) | 0.665 |
|  | TPSR vs HPM | 0.014(-4.6,4.7) | 0.70(-2.1,3.5) | 0.52(-1.8,2.9) | 0.795 |

**Supplementary Table S4. Relative Effects for Decision-Making.**

| Model | CON | SE | TGfU | CL | HPM | TPSR |
| --- | --- | --- | --- | --- | --- | --- |
| CON | — | 0.51 (-1.48, 2.52) | 0.83 (0.36, 1.31) | 0.67 (-1.35, 2.68) | 1.41 (-0.06, 2.86) | 0.86 (-1.58, 3.29) |
| SE | -0.51 (-2.52, 1.48) | — | 0.32 (-1.73, 2.37) | 0.15 (-2.74, 3.00) | 0.91 (-1.62, 3.37) | 0.33 (-2.85, 3.47) |
| TGfU | -0.83 (-1.31, -0.36) | -0.32 (-2.37, 1.73) | — | -0.16 (-2.23, 1.91) | 0.58 (-0.81, 1.97) | 0.03 (-2.35, 2.41) |
| CL | -0.67 (-2.68, 1.35) | -0.15 (-3.00, 2.74) | 0.16 (-1.91, 2.23) | — | 0.74 (-1.74, 3.21) | 0.18 (-2.96, 3.32) |
| HPM | -1.41 (-2.86, 0.06) | -0.91 (-3.37, 1.62) | -0.58 (-1.97, 0.81) | -0.74 (-3.21, 1.74) | — | -0.56 (-2.51, 1.41) |
| TPSR | -0.86 (-3.29, 1.58) | -0.33 (-3.47, 2.85) | -0.03 (-2.41, 2.35) | -0.18 (-3.32, 2.96) | 0.56 (-1.41, 2.51) | — |

Note, Values are standardized mean differences (row vs. column) with 95% credible intervals (CrI).

**Supplementary Table S5. Relative Effects for Skill Execution.**

| Model | CON | SE | TGfU | CL | HPM | TPSR |
| --- | --- | --- | --- | --- | --- | --- |
| **CON** | — | -1.53 (-2.95, -0.11) | -0.31 (-2.93, 2.35) | -1.05 (-5.51, 3.51) | -1.32 (-2.88, 0.22) | -0.80 (-2.77, 1.20) |
| **SE** | 1.53 (0.11, 2.95) | — | 1.24 (-1.73, 4.19) | 0.47 (-4.21, 5.24) | -0.22 (-1.88, 2.28) | 0.73 (-1.68, 3.17) |
| **TGfU** | 0.31 (-2.35, 2.93) | -1.24 (-4.19, 1.73) | — | 0.76 (-5.90, 4.55) | 1.01 (-3.79, 1.78) | 0.51 (-3.76, 2.78) |
| **CL** | 1.05 (-3.51, 5.51) | -0.47 (-5.24, 4.21) | -0.76 (-4.55, 5.90) | — | 0.27 (-4.50, 5.11) | 0.28 (-5.14, 5.14) |
| **HPM** | 1.32 (-0.22, 2.88) | 0.22 (-2.28, 1.88) | -1.01 (-1.78, 3.79) | -0.27 (-5.11, 4.50) | — | -0.51 (-1.82, 2.88) |
| **TPSR** | 0.80 (-1.20, 2.77) | -0.73 (-3.17, 1.68) | -0.51 (-2.78, 3.76) | -0.28 (-5.14, 5.14) | 0.51 (-2.88, 1.82) | — |

**Supplementary Table S6. Relative Effects for Motivation.**

| Model | CON | SE | TGfU | CL | HPM | TPSR |
| --- | --- | --- | --- | --- | --- | --- |
| **CON** | — | -0.48 (-2.31, 1.39) | -0.62 (-2.74, 1.55) | -0.55 (-2.88, 1.77) | -0.96 (-2.63, 0.74) | -0.51 (-2.66, 1.66) |
| **SE** | 0.48 (-1.39, 2.31) | — | -0.14 (-2.39, 2.09) | -0.07 (-2.54, 2.39) | -0.48 (-2.32, 1.34) | -0.03 (-2.34, 2.29) |
| **TGfU** | 0.62 (-1.55, 2.74) | 0.14 (-2.09, 2.39) | — | 0.07 (-2.61, 2.73) | -0.34 (-2.37, 1.68) | 0.11 (-2.46, 2.62) |
| **CL** | 0.55 (-1.77, 2.88) | 0.07 (-2.39, 2.54) | -0.07 (-2.73, 2.61) | — | -0.41 (-2.69, 1.86) | 0.04 (-2.70, 2.76) |
| **HPM** | 0.96 (-0.74, 2.63) | 0.48 (-1.34, 2.32) | 0.34 (-1.68, 2.37) | 0.41 (-1.86, 2.69) | — | 0.45 (-1.66, 2.55) |
| **TPSR** | 0.51 (-1.66, 2.66) | 0.03 (-2.29, 2.34) | -0.11 (-2.62, 2.46) | -0.04 (-2.76, 2.70) | -0.45 (-2.55, 1.66) | — |

**Supplementary Table S7. Relative Effects for Enjoyment.**

| Model | CON | SE | TGfU | CL | HPM | TPSR |
| --- | --- | --- | --- | --- | --- | --- |
| **CON** | — | -0.42 (-1.86, 1.01) | -0.37 (-1.92, 1.20) | -0.28 (-1.74, 1.19) | -0.71 (-2.02, 0.61) | -0.33 (-1.85, 1.19) |
| **SE** | 0.42 (-1.01, 1.86) | — | 0.05 (-1.62, 1.72) | 0.14 (-1.52, 1.82) | -0.29 (-1.72, 1.13) | 0.09 (-1.63, 1.80) |
| **TGfU** | 0.37 (-1.20, 1.92) | -0.05 (-1.72, 1.62) | — | 0.09 (-1.66, 1.83) | -0.34 (-1.82, 1.13) | 0.04 (-1.73, 1.80) |
| **CL** | 0.28 (-1.19, 1.74) | -0.14 (-1.82, 1.52) | -0.09 (-1.83, 1.66) | — | -0.43 (-1.97, 1.10) | -0.05 (-1.83, 1.72) |
| **HPM** | 0.71 (-0.61, 2.02) | 0.29 (-1.13, 1.72) | 0.34 (-1.13, 1.82) | 0.43 (-1.10, 1.97) | — | 0.38 (-1.12, 1.88) |
| **TPSR** | 0.33 (-1.19, 1.85) | -0.09 (-1.80, 1.63) | -0.04 (-1.80, 1.73) | 0.05 (-1.72, 1.83) | -0.38 (-1.88, 1.12) | — |

**Supplementary Table S8. Number and heterogeneity of comparisons across PMs.**

| Comparison | Decision making | | Skill execution | | motivation | | enjoyment | |
| --- | --- | --- | --- | --- | --- | --- | --- | --- |
|  | Number | I^2^. pari | Number | I^2^. pari | Number | I^2^. pari | Number | I^2^. pari |
|  | 23 | 89.0% | 44 |  | 37 | 82.5% | 26 | 99.8% |
| SE vs. CON | 1 | - | 9 | 83.1% | 15 | 86.2% | 10 | 99.9% |
| TGfU vs. CON | 18 | 89.7% | 26 | 87.3% | 4 | 88.5% | 2 | - |
| CL vs CON | 1 | - | 3 | 58.7% | 3 | 88.6% | 1 | - |
| HPM vs. CON | - | - | 3 | 0.00 | 7 | 71.9% | 7 | 98.7% |
| HPM vs. TGfU | 2 | 0.00 | 3 | 64.8% | 1 | - | 1 | 0.000 |
| TPSR vs. CON | - | - | - | - | 7 | 50.8% | 4 | 91.6% |
| HPM vs. TPSR | 1 | - | - | - | - | - | 1 | - |


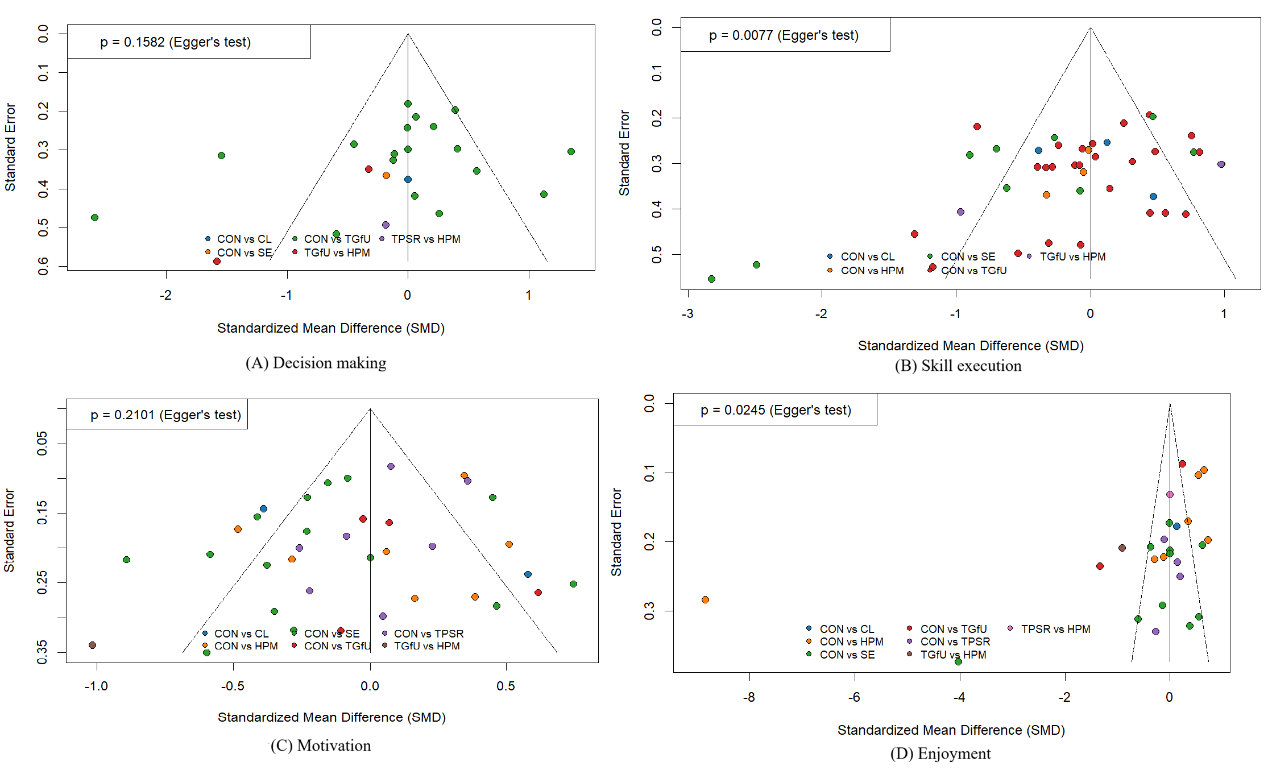


**Supplementary Figure S2. Funnel plot of learning outcomes.**

**References**

1. Alcalá, D. H., & Garijo, A. H. (2017). Teaching games for understanding: A comprehensive approach to promote student’s motivation in physical education. *Journal of human kinetics*, *59*, 17.
2. Altınkök, M. (2017). The effect of movement education based on cooperative learning method on the development of basic motor skills of primary school 1st grade learners. *Journal of Baltic Science Education*, *16*(2), 241-249.
3. Amado, D., Del Villar, F., Leo, F. M., Sánchez-Oliva, D., Sánchez-Miguel, P. A., & García-Calvo, T. (2014). Effect of a multi-dimensional intervention programme on the motivation of physical education students. *PloS one*, *9*(1), e85275.
4. Andrianto, J. R. (2023). Teaching games for understanding (TGfU) learning model on learning motivation in soccer learning. *JOURNAL RESPECS (Research Physical Education and Sports)*, *5*(2), 296-300.
5. Ben Khalifa, W., Zouaoui, M., Zghibi, M., & Azaiez, F. (2020). Effects of verbal interactions between students on skill development, game performance and game involvement in soccer learning. *Sustainability*, *13*(1), 160.
6. Buendía, Á. G., Martínez, B. J. S. A., Izquierdo, M. I. C., & Mármol, A. G. (2022). Effects of a hybrid teaching model (SEM+ TGfU) and the model of personal and social responsibility on sportsmanship and enjoyment in 4 Secondary and 1 Baccalaureate students. *Retos: nuevas tendencias en educación física, deporte y recreación*(43), 550-559.
7. Cecchini Estrada, J. A., González González-Mesa, C., Llamedo, R., Sánchez Martínez, B., & Rodríguez Pérez, C. (2019). The impact of cooperative learning on peer relationships, intrinsic motivation and future intentions to do sport. *Psicothema, 31*.
8. Cecchini, J. A., Montero, J., Alonso, A., Izquierdo, M., & Contreras, O. (2007). Effects of personal and social responsibility on fair play in sports and self-control in school-aged youths. *European Journal of sport science*, *7*(4), 203-211.
9. Cecchini, J. A., Montero, J., & Peña, J. V. (2003). Repercusiones del programa de intervención para desarrollar la responsabilidad personal y social de Hellison sobre los comportamientos de fair-play y el auto-control. *Psicothema*, *15*(4), 631-637.
10. Chang, Y.-K., Chen, S., Tu, K.-W., & Chi, L.-K. (2016). Effect of autonomy support on self-determined motivation in elementary physical education. *Journal of sports science & medicine*, *15*(3), 460.
11. Chatzipanteli, A., Digelidis, N., & Papaioannou, A. G. (2015). Self-regulation, motivation and teaching styles in physical education classes: An intervention study. *Journal of Teaching in Physical Education*, *34*(2), 333-344.
12. Chatzopoulos, D., Drakou, A., Kotzamanidou, M., & Tsorbatzoudis, H. (2006). Girls' soccer performance and motivation: games vs technique approach. *Perceptual and motor skills*, *103*(2), 463-470.
13. Chenchen, X., Rong, G., & Shuaijing, X. (2019). Impact of a sport education season on students' table tennis skills and attitudes in China's high school. *International Journal of Information and Education Technology*, *9*(11), 820-825.
14. Choi, S. M., Sum, K. W. R., Leung, F. L. E., Wallhead, T., Morgan, K., Milton, D.,…Sit, H. P. C. (2021). Effect of sport education on students’ perceived physical literacy, motivation, and physical activity levels in university required physical education: a cluster-randomized trial. *Higher Education*, *81*, 1137-1155.
15. Chu, Y., Chen, C., Wang, G., & Su, F. (2022). The effect of education model in physical education on student learning behavior. *Frontiers in Psychology*, *13*, 944507.
16. Cuevas, R., García-López, L. M., & Contreras, O. (2015). Influence of the Sport Education Model in the psychological basic needs.
17. Cuevas, R., García-López, L. M., & Serra-Olivares, J. (2016). Sport education model and self-determination theory: An intervention in secondary school children. *Kinesiology*, *48*(1.), 30-38.
18. Darnis, F., & Lafont, L. (2015). Cooperative learning and dyadic interactions: Two modes of knowledge construction in socio-constructivist settings for team-sport teaching. *Physical Education and Sport Pedagogy*, *20*(5), 459-473.
19. Ebrahimi, S., & Hasan, M. (2025). Asserting the Impact of Hybrid and Sgtsp Approaches on Students’ Knowledge and Skills in Volleyball: A Comprehensive Analysis. *Available at SSRN 5272743*.
20. Fernández-Río, J., Cecchini, J. A., & Méndez-Giménez, A. (2014). Effects of cooperative learning on perceived competence, motivation, social goals, effort and boredom in prospective Primary Education teachers/Efectos del aprendizaje cooperativo sobre la competencia percibida, la motivación, las relaciones sociales, el esfuerzo y el aburrimiento de futuros docentes de Educación Primaria. *Journal for the Study of Education and Development*, *37*(1), 57-89.
21. Fernández-Río, J., Méndez-Giménez, A., & Méndez Alonso, D. (2017). Efects of two instructional approaches, sport education and direct instruction, on secondary education students’ psychological response.
22. Fernandez-Rio, J., Sanz, N., Fernandez-Cando, J., & Santos, L. (2017). Impact of a sustained Cooperative Learning intervention on student motivation. *Physical Education and Sport Pedagogy*, *22*(1), 89-105.
23. García-Castejón, G., Camerino, O., Castañer, M., Manzano-Sánchez, D., Jiménez-Parra, J. F., & Valero-Valenzuela, A. (2021). Implementation of a hybrid educational program between the model of personal and social responsibility (TPSR) and the teaching games for understanding (TGfU) in physical education and its effects on health: an approach based on mixed methods. *Children*, *8*(7), 573.
24. Gil-Arias, A., Harvey, S., Cárceles, A., Práxedes, A., & Del Villar, F. (2017). Impact of a hybrid TGfU-Sport Education unit on student motivation in physical education. *PloS one*, *12*(6), e0179876.
25. Gil-Arias, A., Harvey, S., García-Herreros, F., González-Víllora, S., Práxedes, A., & Moreno, A. (2021). Effect of a hybrid teaching games for understanding/sport education unit on elementary students’ self-determined motivation in physical education. *European Physical Education Review*, *27*(2), 366-383.
26. Gil, V. M. G., Álvarez, F. D. V., Pizarro, A. P., & Domínguez, A. M. (2019). El cuestionamiento como herramienta fundamental para el desarrollo de la toma de decisiones de los alumnos en educación física. *Movimento*, *25*, e25028.
27. Gray, S., & Sproule, J. (2011). Developing pupils’ performance in team invasion games. *Physical Education and Sport Pedagogy*, *16*(1), 15-32.
28. Guijarro-Romero, S., Mayorga-Vega, D., & Viciana, J. (2018). Aprendizaje táctico en deportes de invasión en la educación física: Influencia del nivel inicial de los estudiantes. *Movimento*, *24*, 889-902.
29. Hastie, P. A., Calderón, A., Rolim, R. J., & Guarino, A. J. (2013). The development of skill and knowledge during a sport education season of track and field athletics. *Research Quarterly for Exercise and Sport*, *84*(3), 336-344.
30. Hernández-Andreo, L., Gómez-Mármol, A., & Cifo-Izquierdo, M. I. (2020). Effects on motivation and implicit beliefs about self ability using the Sports Education Model and the traditional style in secondary education. *Sustainability*, *12*(9), 3843.
31. Lemus, I. L., Pizarro, A. P., & Álvarez, F. V. (2016). Effect of an intervention teaching program, based on TGFU model, on the cognitive and execution variables, in the physical education context. *European Journal of Human Movement*(37), 88-108.
32. Luo, Y.-J., Lin, M.-L., Hsu, C.-H., Liao, C.-C., & Kao, C.-C. (2020). The effects of team-game-tournaments application towards learning motivation and motor skills in college physical education. *Sustainability*, *12*(15), 6147.
33. Manzano-Sánchez, D. (2023). GAMIFICATION AND COOPERATIVE LEARNING: EFFECTS OF A HYBRIDIZATION IN PHYSICAL EDUCATION GAMIFICACIÓN Y APRENDIZAJE COOPERATIVO: EFECTOS DE UNA HIBRIDACIÓN EN EDUCACIÓN. *Revista Internacional de Medicina y Ciencias de la Actividad*, *23(91)*, 321-342.
34. Manzano-Sánchez, D., & Gómez-López, M. (2023). Personal and social responsibility model: Differences according to educational stage in motivation, basic psychological needs, satisfaction, and responsibility. *Children*, *10*(5), 864.
35. Manzano-Sánchez, D., González-Víllora, S., & Valero-Valenzuela, A. (2021). Application of the Teaching Personal and Social Responsibility model in the secondary education curriculum: Implications in psychological and contextual variables in students. *International Journal of Environmental Research and Public Health*, *18*(6), 3047.
36. Manzano-Sánchez, D., & Valero-Valenzuela, A. (2019). Implementation of a model-based programme to promote personal and social responsibility and its effects on motivation, prosocial behaviours, violence and classroom climate in primary and secondary education. *International Journal of Environmental Research and Public Health*, *16*(21), 4259.
37. Manzano-Sánchez, D., Valero-Valenzuela, A., Conde-Sánchez, A., & Chen, M.-Y. (2019). Applying the personal and social responsibility model-based program: Differences according to gender between basic psychological needs, motivation, life satisfaction and intention to be physically active. *International Journal of Environmental Research and Public Health*, *16*(13), 2326.
38. Medina-Casaubón, J., & Burgueño, R. (2017). Influencia de una temporada de educacón deportiva sobre las estrategias motivacionales en alumnado de bachillerato: Una visión desde la teoría de la auto-determinación.
39. Melero, D., Manzano Sánchez, D., Navarro Ardoy, D., Morales Baños, V., & Valero Valenzuela, A. (2021). The Seneb's Enigma: Impact of a Hybrid Personal and Social Responsibility and Gamification Model-Based Practice on Motivation and Healthy Habits in Physical Education.
40. Menickelli, J., & Hastie, P. A. (2014). The impact of two curricular models on motivation, engagement and achievement in physical education. *International Journal of Physical Education, Fitness and Sports*, *3*(2), 33-42.
41. Merino-Barrero, J. A., Valero-Valenzuela, A., Pedreno, N. B., & Fernandez-Río, J. (2019). Impact of a sustained TPSR program on students’ responsibility, motivation, sportsmanship, and intention to be physically active. *Journal of Teaching in Physical Education*, *39*(2), 247-255.
42. Miller, A., Christensen, E., Eather, N., Gray, S., Sproule, J., Keay, J., & Lubans, D. (2016). Can physical education and physical activity outcomes be developed simultaneously using a game-centered approach? *European Physical Education Review*, *22*(1), 113-133.
43. Morales-Belando, M. T., & Arias-Estero, J. L. (2017). Effect of teaching races for understanding in youth sailing on performance, knowledge, and adherence. *Research Quarterly for Exercise and Sport*, *88*(4), 513-523.
44. Nathan, S. (2016). Badminton instructional in Malaysian schools: a comparative analysis of TGfU and SDT pedagogical models. *SpringerPlus*, *5*(1), 1215.
45. Nathan, S., & Haynes, J. (2013). A move to an innovative games teaching model: Style E Tactical (SET). *Asia-Pacific Journal of Health, Sport and Physical Education*, *4*(3), 287-302.
46. Navarro-Patón, R., Basanta-Camiño, S., & Gómez, C. A. (2017). Cooperative games: Incidence in motivation, basic psychological needs and enjoyment in Primary School. *Sport. Sci. Tech. J. Sch. Sport Phys. Educ. Psychomot*, *3*, 589-604.
47. Osman, A. (2017). Effects of teaching games for understanding on tactical awareness and decision making in soccer for college students. *Science, Movement and Health*, *17*(2), 170-176.
48. Pan, Y.-H., Huang, C.-H., & Hsu, W.-T. (2023). A comparison of the learning effects between TGfU-SE and TGfU on learning motivation, sport enjoyment, responsibility, and game performance in physical education. *Frontiers in Psychology*, *14*, 1165064.
49. Pan, Y.-H., Huang, C.-H., Lee, I.-S., & Hsu, W.-T. (2019). Comparison of learning effects of merging TPSR respectively with sport education and traditional teaching model in high school physical education classes. *Sustainability*, *11*(7), 2057.
50. Pereira, J., Araújo, R., Farias, C., Bessa, C., & Mesquita, I. (2016). Sport education and direct instruction units: Comparison of student knowledge development in athletics. *Journal of sports science & medicine*, *15*(4), 569.
51. Perlman, D. (2010). Change in affect and needs satisfaction for amotivated students within the sport education model. *Journal of Teaching in Physical Education*, *29*(4), 433-445.
52. Perlman, D. (2012). The influence of the sport education model on developing autonomous instruction. *Physical Education and Sport Pedagogy*, *17*(5), 493-505.
53. Perlman, D. J. (2011). Examination of self-determination within the sport education model. *Asia-Pacific Journal of Health, Sport and Physical Education*, *2*(1), 79-92.
54. Práxedes, A., Del Villar, F., Pizarro, D., & Moreno, A. (2018). The impact of nonlinear pedagogy on decision-making and execution in youth soccer players according to game actions. *Journal of human kinetics*, *62*(1), 185-198.
55. Práxedes, A., Moreno, A., Sevil, J., García-González, L., & Del Villar, F. (2016). A preliminary study of the effects of a comprehensive teaching program, based on questioning, to improve tactical actions in young footballers. *Perceptual and motor skills*, *122*(3), 742-756.
56. Pritchard, T., Hawkins, A., Wiegand, R., & Metzler, J. N. (2008). Effects of two instructional approaches on skill development, knowledge, and game performance. *Measurement in Physical Education and Exercise Science*, *12*(4), 219-236.
57. Psotta, R., & Martin, A. (2011). Changes in decision-making skill and skill execution in soccer performance: The intervention study. *Acta Gymnica*, *41*(2), 7-15.
58. Sánchez, D. M., González-Víllora, S., & Valenzuela, A. V. (2021). Application of teaching personal and social responsibility model to the secondary education curriculum. Implications for students and teachers.
59. Shariati, S., Nazari, S., Norouzi Seyed Hossini, R., Manzano-Sánchez, D., & Norouzi, E. (2024). Hybrid Pedagogical Intervention can Decrease Impulsivity and Antisocial Behavior and Improve Motor and Cognitive Functions Among Iranian Adolescent. *Canadian Journal of School Psychology*, *39*(3), 266-285.
60. Sierra-Ríos, J. V., Clemente, F. M., Rey, E., & González-Víllora, S. (2020). Effects of 6 weeks direct instruction and teaching games for understanding programs on physical activity and tactical behaviour in U-12 soccer players. *International Journal of Environmental Research and Public Health*, *17*(14), 5008.
61. Spittle, M., & Byrne, K. (2009). The influence of sport education on student motivation in physical education. *Physical Education and Sport Pedagogy*, *14*(3), 253-266.
62. Viciana, J., Casado-Robles, C., Pérez-Macías, L., & Mayorga-Vega, D. (2020). A Sport Education teaching unit as a citizenship education strategy in Physical Education. A group-randomized controlled trial. *Retos: Nuevas Perspectivas de Educación Física, Deporte y Recreación*, *38*.
63. Wallhead, T. L., Garn, A. C., & Vidoni, C. (2014). Effect of a sport education program on motivation for physical education and leisure-time physical activity. *Research Quarterly for Exercise and Sport*, *85*(4), 478-487.
64. Wallhead, T. L., & Ntoumanis, N. (2004). Effects of a sport education intervention on students’ motivational responses in physical education. *Journal of Teaching in Physical Education*, *23*(1), 4-18.
65. Yang, C., & Lu, P. (2013). The experimental study of teaching games for understanding in college football teaching. 2013 International Workshop on Computer Science in Sports,
